# Tislelizumab efficacy and safety compared to other anti–PD-1s: a network meta-analysis of first-line therapies for unresectable, locally advanced or metastatic esophageal squamous cell carcinoma

**DOI:** 10.3389/fimmu.2025.1657085

**Published:** 2026-01-06

**Authors:** Jaffer A. Ajani, Elizabeth Smyth, David Tougeron, Hyun Ae Jung, Wenxi Tang, Jason Steenkamp, Emily Prentiss, JeanPierre Coaquira Castro, Kirk Szafranski, Lin Zhan

**Affiliations:** 1 Department of Gastrointestinal Medical Oncology, The University of Texas MD Anderson Cancer Center, Houston, TX, United States; 2 Department of Oncology, Oxford University Hospitals NHS Foundation Trust, Oxford, United Kingdom; 3 Department of Gastroenterology and Hepatology, Poitiers University Hospital, Poitiers, France; 4 Division of Hematology-Oncology, Department of Medicine, Samsung Medical Center, Sungkyunkwan University School of Medicine, Seoul, Republic of Korea; 5 BeOne Medicines, Ltd., San Carlos, CA, United States; 6 Department of Value and Evidence, EVERSANA, Burlington, ON, Canada

**Keywords:** esophageal squamous cell carcinoma, immunotherapies, tislelizumab, systematic literature review, network meta-analysis, indirect treatment comparison

## Abstract

**Introduction:**

The addition of programmed cell death protein-1 (PD-1) inhibitors to chemotherapy (CT) or anti-CTLA4 (ipilimumab) has recently emerged as an effective first-line (1L) treatment for esophageal squamous cell carcinoma (ESCC), the most common form of esophageal cancer globally.

**Methods:**

A systematic literature review (SLR) was conducted to identify randomized controlled trials (RCTs) investigating 1L PD-1 inhibitor regimens in adult patients with unresectable, locally advanced, or metastatic ESCC. Bayesian NMAs were conducted to evaluate overall survival (OS), progression-free survival (PFS), objective response rate (ORR), and grade ≥3 treatment-related adverse events (TRAEs).

**Results:**

Three eligible RCTs were identified, evaluating three PD-1 inhibitor regimens with broad regulatory approval for 1L ESCC in combination with CT (tislelizumab, nivolumab, and pembrolizumab). Tislelizumab + CT demonstrated similar long-term OS to nivolumab + CT and pembrolizumab + CT but a significant PFS benefit over nivolumab + CT and comparable efficacy to pembrolizumab + CT. Subgroup analyses were consistent with the base case, including among patients with varying PD-L1 expression (≥1% and ≥5% Tumor Area Positivity [TAP] score or ≥1 and ≥5 combined positive score [CPS]), Asia versus the rest of world, and different underlying CT backbones. Safety profiles were comparable across the three treatments.

**Conclusion:**

Tislelizumab + CT is an effective 1L treatment option for advanced or metastatic ESCC, demonstrating comparable efficacy and safety outcomes relative to existing treatments.

## 1 Introduction

Globally, esophageal squamous cell carcinoma (ESCC) is the most common form of esophageal cancer, comprising approximately 85-90% of all cases worldwide, with the remaining cases being attributed to esophageal adenocarcinoma (EAC) (1, 2), of which approximately 50% present as advanced or metastatic, unresectable disease at diagnosis (3–6). Patients with advanced ESCC face a particularly poor prognosis, with a median estimated overall survival of less than 1 year (7, 8), and 5-year survival rates typically ranging between 10% and 30% (1, 9, 10).

Combining programmed cell death protein-1 (PD-1) inhibitors with chemotherapy (CT) has recently emerged as a promising first-line (1L) treatment to enhance outcomes for patients with advanced or metastatic ESCC who are eligible for initial systemic therapy (11). To date, broad regulatory approval has been granted for 1L treatment with tislelizumab + CT, pembrolizumab + CT, and nivolumab + CT for select patients with positive expression for programmed death-ligand 1 (PD-L1), including by the United States (US) Food and Drug Administration and the European Medicines Agency (12–20). The minimum PD-L1 expression threshold varies across approved 1L indications. Tislelizumab + CT is approved for use in patients whose tumors express PD-L1 ≥1 in the US and a PD-L1 TAP score ≥5% in the European Union (EU), respectively (13, 15). Based on the phase 3 KEYNOTE-590 trial (NCT03189719), pembrolizumab + CT is approved in the US for patients with tumors with a CPS ≥1, and in the EU for those with tumors with a CPS ≥10 (17, 20). Similarly, the phase 3 CheckMate-648 trial (NCT03143153) led to the approval of both nivolumab + CT and nivolumab + ipilimumab in the US for patients whose tumors express PD-L1 (≥1), whereas in the EU, these regimens are restricted for use in patients with a tumor proportion score (TPS) ≥1% (18, 19).

In the randomized, phase 3 RATIONALE-306 trial (NCT03783442), tislelizumab + CT demonstrated more favorable overall survival (OS) outcomes and a manageable safety profile relative to placebo + CT as 1L treatment of advanced or metastatic ESCC in the intent-to-treat (ITT) population, with the greatest OS benefit being observed in patients with a tumor PD-L1 TAP score ≥10% (secondary endpoint analysis) (21). Further, a recent retrospective analysis of RATIONALE-306 also found that tislelizumab + CT was associated with a clinically meaningful improvement in OS compared with placebo + CT among patients with advanced or metastatic, unresectable ESCC and tumor PD-L1 ≥1 at primary analysis (22). There is no standardized methodology for PD-L1 testing across currently indicated PD-1 therapies for ESCC, with testing methods varying regionally by local clinical practices, although there is a relatively high level of concordance between TAP score versus CPS (overall percentage agreement [OPA]: 90%) and TPS (OPA: 78%) (23).

Tislelizumab + CT has not been directly compared with pembrolizumab + CT, nivolumab + CT or ipilimumab, or other PD-1 inhibitor–based immuno-oncology (IO) regimens for the 1L treatment of ESCC. The present study is a network meta-analysis (NMA) conducted to assess the comparative efficacy and safety of tislelizumab + CT versus broadly approved 1L IO regimens for patients with unresectable, locally advanced or metastatic ESCC.

## 2 Methods

### 2.1 Systematic literature review

A systematic literature review (SLR) was conducted to identify and summarize published data on 1L IO treatments in adult patients with unresectable, locally advanced or metastatic ESCC. The review was performed in accordance with the Cochrane Handbook for Systematic Reviews of Interventions and reported per the Preferred Reporting Items for Systematic Literature reviews and Meta-analyses (PRISMA) statement (24–26). The protocol of the SLR was not registered. The search strategy was developed and tested through an iterative process by a medical information specialist in consultation with the review team, then peer-reviewed independently by another senior medical information specialist. Please refer to **Appendix A.1** of the **Supplementary Material** for the fully detailed search strategy. The search was conducted on June 23, 2023, using the Ovid^®^ platform to search the following electronic databases: Embase^®^, Ovid MEDLINE^®^ (including Epub Ahead of Print and In-Process & Other Non-Indexed Citations), Ovid MEDLINE^®^ Daily, Cochrane Central Register of Controlled Trials, and the Cochrane Database of Systematic Reviews.

The search was limited to randomized controlled trials (RCTs), SLRs, and meta-analyses conducted in adults aged 18 years or older. Only publicly available and peer-reviewed data were included. No date restrictions were applied to full-text publications, whereas relevant conference abstracts were included for the past 2 years. Additional searches of conference proceedings, health technology assessment agencies, and trial registries were also performed. Studies included in the SLR investigated a 1L IO therapy alone or in combination with CT or another therapy, with results reported for at least one clinical or safety outcome.

Study selection was conducted by two reviewers who independently assessed eligibility using prespecified criteria (**Appendix Table 2** in **Supplementary Material**). Data for studies meeting all inclusion criteria were extracted into a standardized form in Microsoft^®^ Excel (Microsoft Corporation, Seattle, US) by one reviewer and validated by a second reviewer (see **Appendix A** in **Supplementary Material**). In each process, a third independent reviewer was consulted when consensus could not be achieved. A risk of bias assessment of each included trial was also conducted using the NICE Single Technology Appraisal Evidence Submission Checklist (27) for assessment of risk of bias in RCT. Results of the risk assessment for each included RCT are in **Appendix Table 3** in **Supplementary Material**.

### 2.2 NMA

An NMA feasibility assessment was conducted to evaluate clinical heterogeneity across all relevant trials identified in the clinical SLR. Feasibility was confirmed for the following outcomes: OS, progression-free survival (PFS), objective response rate (ORR), and grade ≥3 treatment-related adverse events (TRAEs). The proportional hazards (PH) assumption was also assessed for OS and PFS via log-cumulative hazard plots, Schoenfeld residuals plots, and the Grambsch–Therneau test (28, 29).

NMAs were conducted using a Bayesian framework and performed using R version 3.6.1, Just Another Gibbs Sampler, and WinBUGS (30). Point estimates and 95% credible intervals (CrIs) were modeled for outcomes using Markov Chain Monte Carlo methods. The probability that each treatment was the most efficacious regimen (P-best), the second best, and so on, was assessed. Surface area under the cumulative ranking curve (SUCRA) values were calculated to reflect the relative probability of an intervention being among the best options (31).

For time-to-event outcomes (OS, PFS), the hazard ratio (HR) and its 95% CrIs were calculated for comparisons between treatments. For ORR and grade ≥3 TRAEs, the odds ratio (OR) and its 95% CrI were calculated. Stratified HRs and 95% confidence intervals (CIs) were used when available; otherwise, the unstratified HRs and associated 95% CIs were used. In several instances where specific data were not reported in the publications identified in the SLR, they were retrieved from the US Food and Drug Administration (FDA) Briefing Document on immune checkpoint inhibitors in patients with metastatic or unresectable ESCC (32, 33).

Studies reporting only the number of responders/events or percentage of responses/events had ORs calculated using contingency tables. Observed differences in HR and OR were considered statistically significant if the 95% CrI range did not cross 1. Although the assumptions of random effects NMA models are generally preferred as they are usually more plausible than fixed effects models, between-study heterogeneity could not be estimated in the present study because only one trial connected each intervention in the evidence networks (34). Therefore, NMA was performed using fixed-effects models. Network diagrams were developed to visualize the evidence base for each outcome. To form connected network diagrams, all CT backbone treatments were assumed to be comparable and were pooled together into a single node (35, 36).

### 2.3 Base case, subgroup, and scenario analyses

The base case used the ITT populations for each trial, comparing tislelizumab + CT to broadly used IO + CT regimens for 1L ESCC (i.e., nivolumab + CT and pembrolizumab + CT) (12, 16–18, 37, 38). The analysis included data for tislelizumab + CT and placebo + CT from the RATIONALE-306 trial (data cut-off: February 28, 2022) (21), and the comparators pembrolizumab + CT (KEYNOTE-590 [data cut-off: July 2, 2020]) (39) and nivolumab + CT (CheckMate 648 [data cut-off: January 18, 2021]) (40). The follow-up periods were generally comparable across all three trials at their respective data cut-off dates (RATIONALE-306: median 16.3 months; KEYNOTE-590: median 22.6 months; CheckMate-648: minimum of 12.9 months).

Subgroup analyses were conducted to assess OS, PFS, and ORR at varying PD-L1 expression thresholds, including PD-L1 ≥1 (TAP score 1% or CPS 1) and PD-L1 ≥5 (TAP score 5% or CPS 5). Due to the disproportionately high incidence of ESCC in Asia (1), subgroup analyses were performed by geographic region (Asia and non-Asia [rest of the world; ROW]). Additionally, to confirm the assumption that the underlying CT treatments were equivalent, subgroup analyses were run with platinum + fluoropyrimidine CT treatments only. No subgroup analyses were performed for grade ≥3 TRAEs due to a lack of available data.

A scenario analysis (refer to **Appendix E** in **Supplementary Material**) was also conducted that included nivolumab + ipilimumab (CheckMate 648 [data cut-off: January 18, 2021]) in the network (40). Nivolumab + ipilimumab was excluded from the base case due to the lower grade of evidence supporting its use in 1L ESCC, per ESMO treatment guidelines (i.e., grade I, B for nivolumab + ipilimumab vs. grade I, A for nivolumab + CT) (41). The current study focused on describing base case and subgroup analysis results.

## 3 Results

### 3.1 Study selection

**Figure 1:**
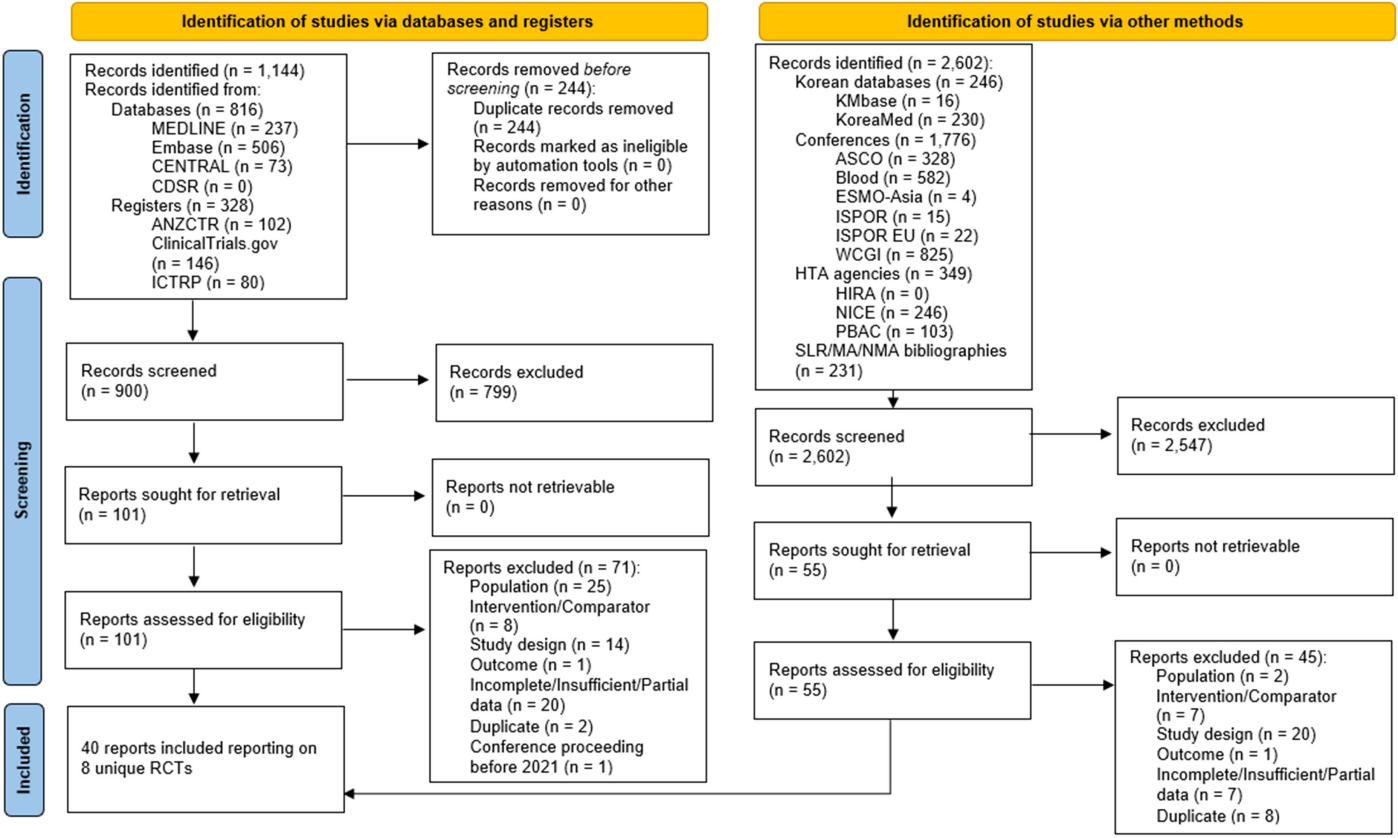
PRISMA flow diagram of clinical evidence. ASCO, American Society of Clinical Oncology; ANZCTR, Australian New Zealand Clinical Trials Registry; CDSR, Cochrane Database of Systematic Reviews; ESMO, European Society for Medical Oncology; HIRA, Health Insurance Review & Assessment Service; HTA, health technology assessment; ICTRP, International Clinical Trials Registry Platform; ISPOR, International Society for Pharmacoeconomics and Outcomes Research; NICE, National Institute for Health and Care Excellence; PBAC, Pharmaceutical Benefits Advisory Committee; PRISMA, Preferred Reporting Items for Systematic reviews and Meta-Analyses; RCT, randomized controlled trial; SLR, systematic literature review; WCGI, World Congress on Gastrointestinal Cancer.

A total of 900 unique records were screened from Ovid^®^ after deduplication, with an additional 2,602 records screened through supplemental searches of conference proceedings, health technology assessment agency websites, and trial registries (**Figure 1**). Following screening, 40 records reporting on eight unique phase 3 RCTs met the eligibility criteria, including four IO regimens with broad regulatory approval (tislelizumab + CT, nivolumab + CT, nivolumab + ipilimumab, and pembrolizumab + CT) and five others with more restricted regulatory approvals (serplulimab, toripalimab, sintilimab, camrelizumab, and sugemalimab; **Appendix Table 4** in **Supplementary Material**) (21, 39, 40, 42–46). In the present NMA, the base case only compared between IO regimens that are currently approved across the EU and US (i.e., tislelizumab, nivolumab, and pembrolizumab), omitting the remaining five regimens that are predominantly only approved in Asia.

The feasibility assessment confirmed the appropriateness of an NMA approach, given the similarity of study populations across eligible trials, as well as the possibility to conduct subgroup analyses and comparisons among all relevant treatments. NMAs were feasible for the following outcomes across three key RCTs (CheckMate 648, RATIONALE-306, and KEYNOTE-590 [1L ESCC subpopulation only]): OS, PFS, ORR, and grade ≥3 TRAEs, under the assumption that all CT backbone treatments are comparable and can be pooled together into a single note.

When considering data from the primary data cut-off for RATIONALE-306 (February 28, 2022), there were no clear violations of the PH assumption among PD-1 inhibitors, except in comparisons involving nivolumab + ipilimumab for OS and PFS. Specifically, the p-values for the Grambsch–Therneau test were <0.05, and patterns suggestive of violation were observed in the log-cumulative hazard and Schoenfeld residual plots (**Appendix F** in **Supplementary Material**). Additional PH assumption violations were also observed for OS between tislelizumab + CT and placebo + CT within the RATIONALE-306 trial. PH assumption testing demonstrated violations in the Grambsch–Therneau test (p < 0.05) for OS, with patterns of violation observed in the log-cumulative hazard and Schoenfeld residual plots (**Appendix G** in **Supplementary Material**). This indicates that the estimated OS and PFS HRs for nivolumab + ipilimumab versus tislelizumab + CT and OS HR for tislelizumab + CT versus placebo + CT should be interpreted with caution. The single HRs for these treatment regimens might not represent the true proportionality of hazard rates across time between the two treatments.

### 3.2 NMA

**Figure 2:**
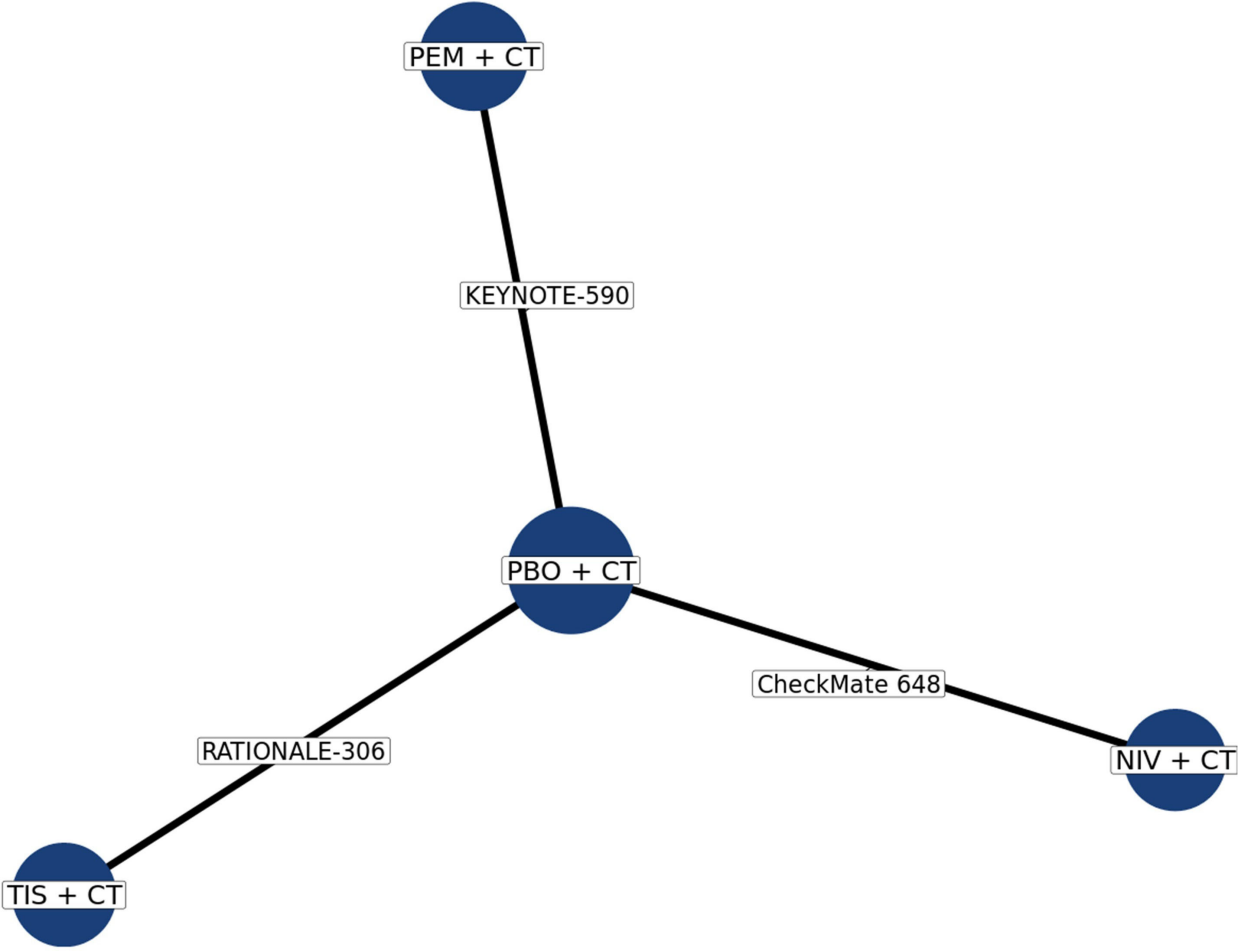
Network diagram for all outcomes in base case. CT, chemotherapy; NIV, nivolumab; PBO, placebo; PEM, pembrolizumab; TIS, tislelizumab.

The base case network consists of four treatment nodes informed by three RCTs (CheckMate 648, RATIONALE-306, and KEYNOTE-590; **Figure 2**); all treatments were anchored to placebo + CT through a single study. The network consisted of 1,842 patients for OS, PFS, and ORR analyses, and 1,999 patients for grade ≥3 TRAEs.

#### 3.2.1 OS, PFS, and ORR

**Figure 3:**
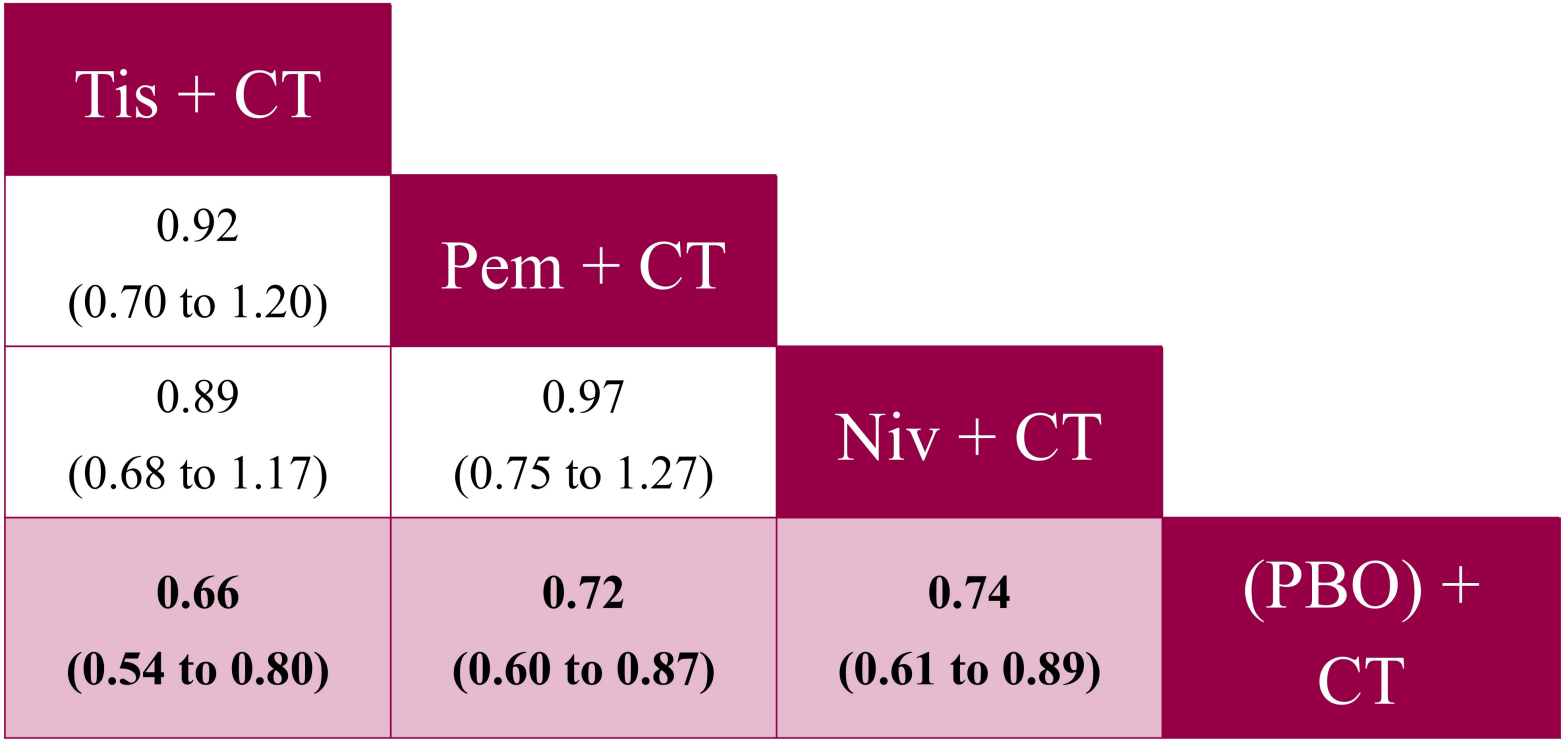
Pairwise comparisons from the fixed effects NMA for OS (reported as HR [95% CrI]) – ITT population analysis. CrI, credible interval; CT, chemotherapy; HR, hazard ratio; ITT, intent-to-treat; niv, nivolumab; NMA, network meta-analysis; OS, overall survival; PBO, placebo; pem, pembrolizumab; tis, tislelizumab.

**Figure 4:**
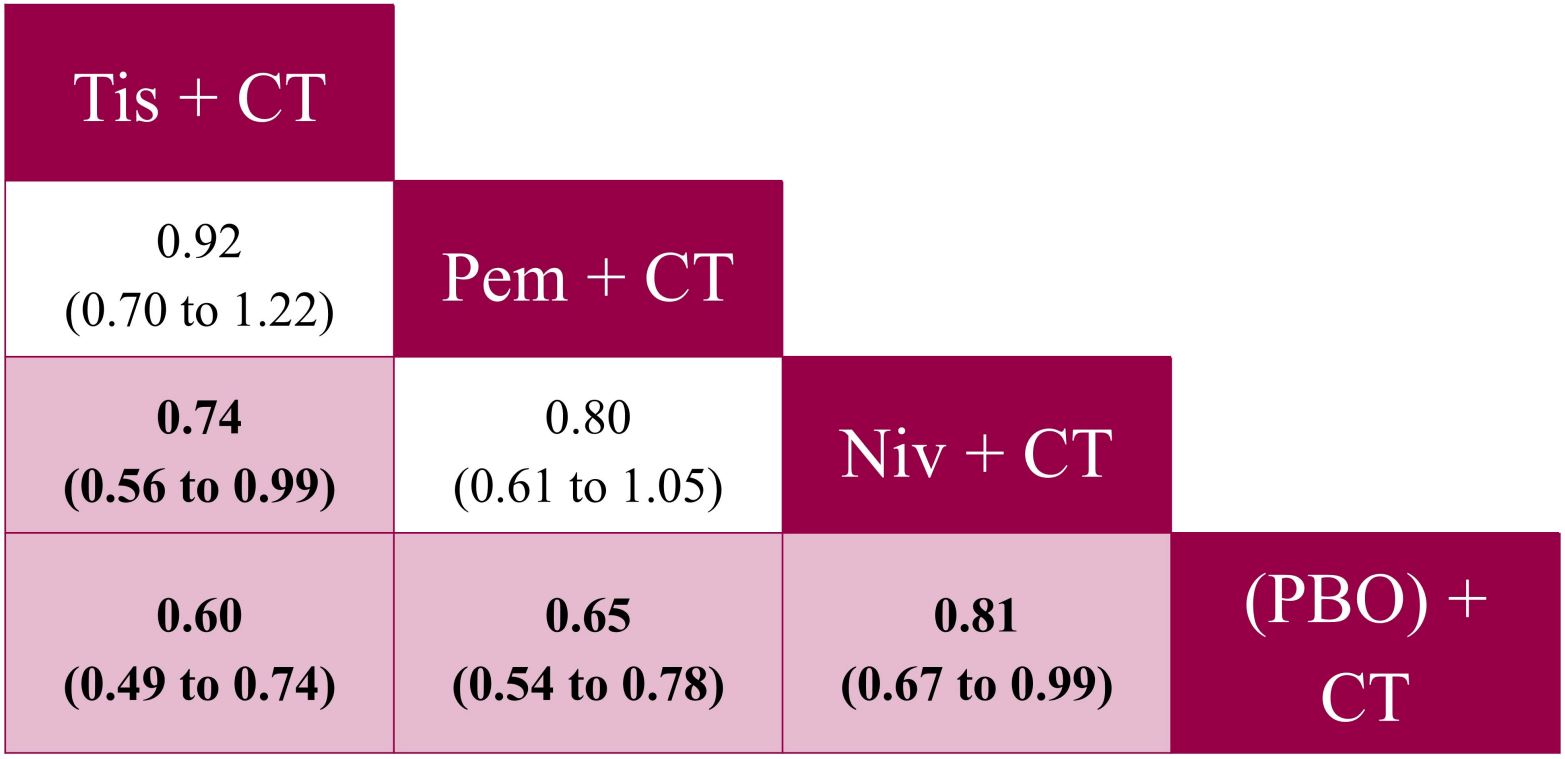
Pairwise comparisons from the fixed effects NMA for PFS (reported as HR [95% CrI]) – ITT population analysis. CrI, credible interval; CT, chemotherapy; HR, hazard ratio; ITT, intent-to-treat; niv, nivolumab; NMA, network meta-analysis; PBO, placebo; pem, pembrolizumab; PFS, progression-free survival; tis, tislelizumab.

**Figure 5:**
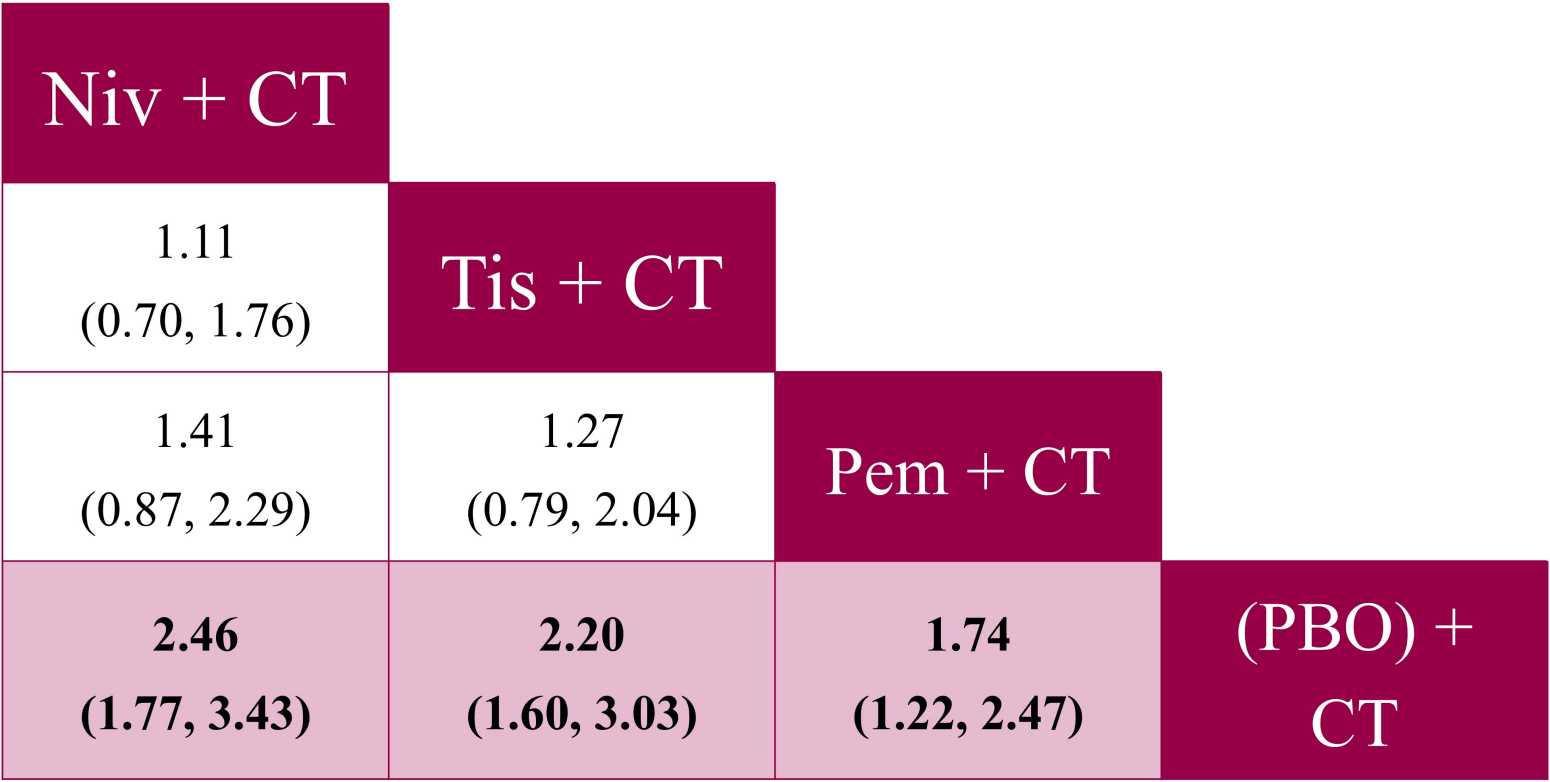
Pairwise comparisons from the fixed effects NMA for ORR (reported as OR [95% CrI]) – ITT population analysis. CrI, credible interval; CT, chemotherapy; ITT, intent-to-treat; niv, nivolumab; NMA, network meta-analysis; OR, odds ratio; ORR, objective response rate; PBO, placebo; pem, pembrolizumab; tis, tislelizumab.

All IO + CT regimens were significantly more favorable to placebo + CT for OS (**Figure 3**), PFS (**Figure 4**), and ORR (**Figure 5**) in the ITT population. For OS, tislelizumab + CT was comparable to both nivolumab + CT (HR: 0.89, 95% CrI: 0.68 to 1.17) and pembrolizumab + CT (HR: 0.92, 95% CrI: 0.70 to 1.20), with no significant differences observed (**Figure 3**). Pembrolizumab + CT also showed a similar OS to nivolumab + CT (HR: 0.97, 95% CrI: 0.75 to 1.27). Tislelizumab + CT was associated with the highest probability of being the most effective treatment (P-best: 64%) and the highest likelihood of being the top-ranked therapy (SUCRA: 84%) (**Appendix Table 5** in **Supplementary Material**).

For PFS, tislelizumab + CT was significantly more favorable to nivolumab + CT (HR: 0.74, 95% CrI: 0.56 to 0.99), and was comparable to pembrolizumab + CT (HR: 0.92, 95% CrI: 0.70 to 1.22) (**Figure 4**). There were also no significant differences observed between pembrolizumab + CT and nivolumab + CT (HR: 0.80, 95% CrI: 0.61 to 1.05). Tislelizumab + CT was associated with the highest SUCRA value of 90% and a P-best score of 71% (**Appendix Table 6** in **Supplementary Material**).

For ORR, tislelizumab + CT was comparable to pembrolizumab + CT (OR: 1.27, 95% CrI: 0.79 to 2.04) and nivolumab + CT (OR for nivolumab + CT vs. tislelizumab + CT: 1.11, 95% CrI: 0.70 to 1.76) (**Figure 5**). Similar outcomes were observed between nivolumab + CT and pembrolizumab + CT (OR: 1.41, 95% CrI: 0.87 to 2.29). Nivolumab + CT was associated with the highest SUCRA value of 87% and P-best score of 65% (**Appendix Table 7** in **Supplementary Material**).

**Figure 6:**
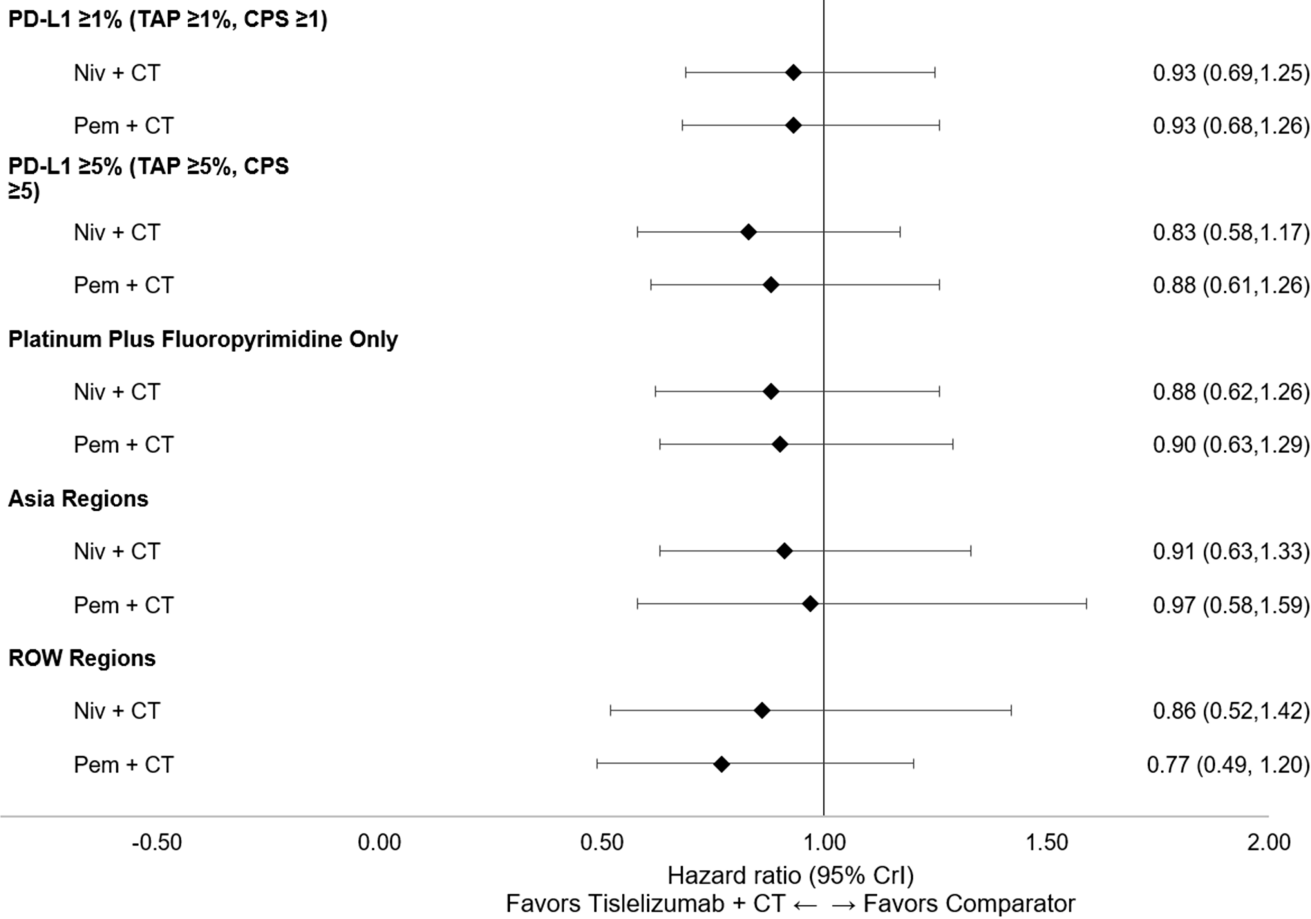
Forest plot of subgroup analyses for OS (reported as HR [95% CrI]). CPS, combined positive score; CrI, credible interval; CT, chemotherapy; HR, hazard ratio; niv, nivolumab; OS, overall survival; PD-L1, programmed death-ligand 1; pem, pembrolizumab; ROW, rest of the world; TAP, Tumor Area Positivity.

**Figure 7:**
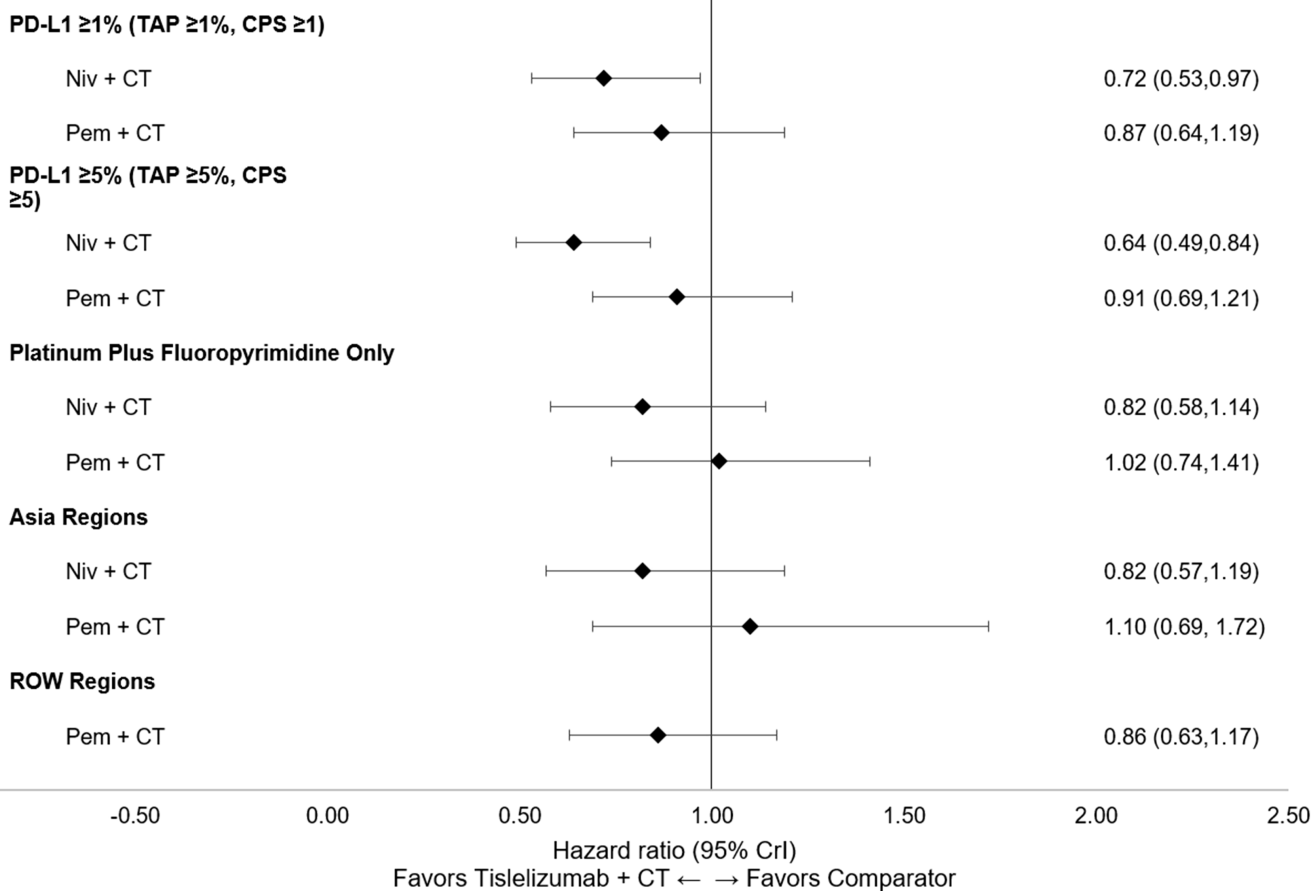
Forest plot of subgroup analyses for PFS (reported as HR [95% CrI]). CPS, combined positive score; CrI, credible interval; CT, chemotherapy; HR, hazard ratio; niv, nivolumab; PD-L1, programmed death-ligand 1; pem, pembrolizumab; PFS, progression-free survival; ROW, rest of the world; TAP, Tumor Area Positivity.

**Figure 8:**
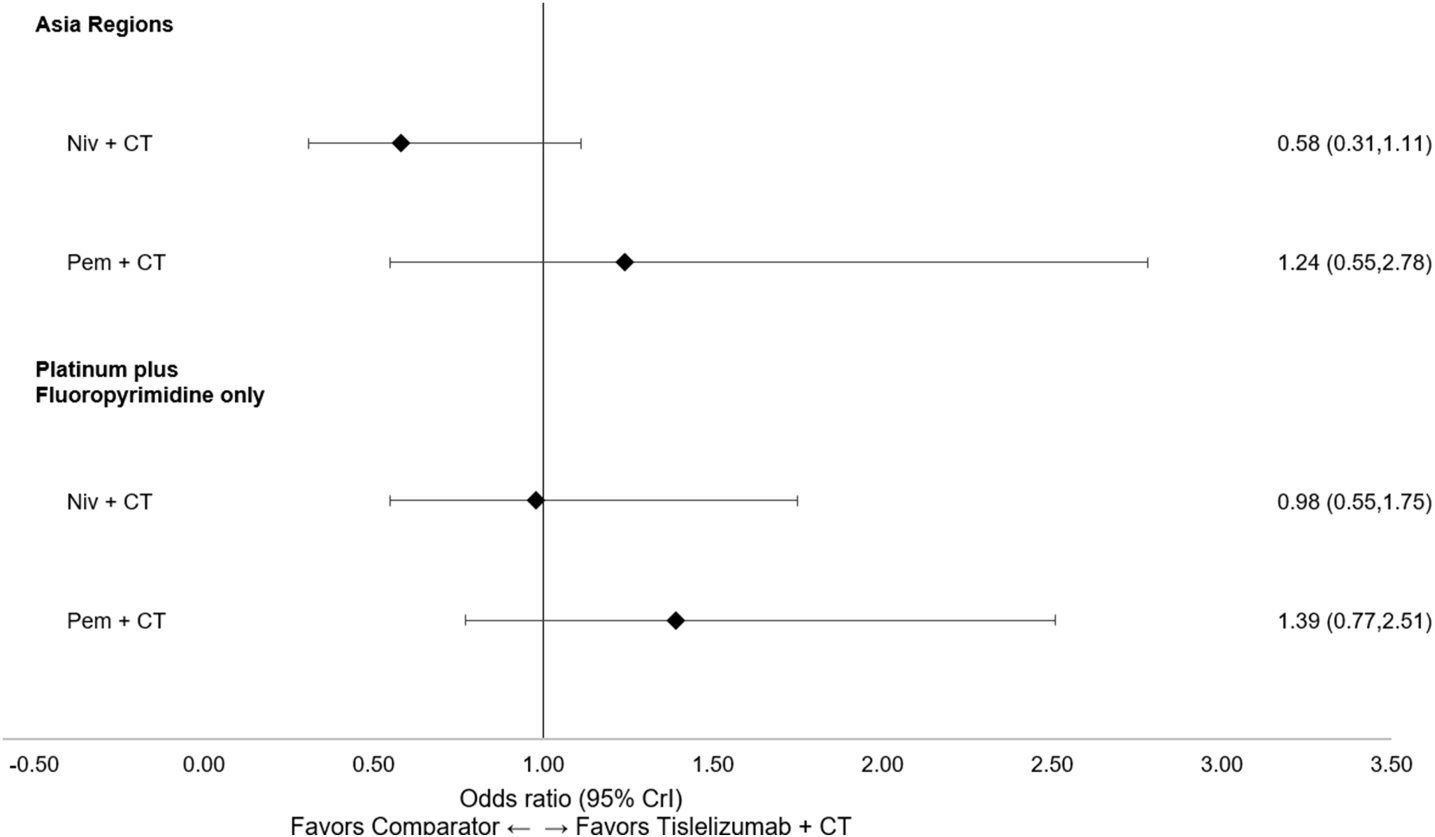
Forest plot of subgroup analyses for ORR (reported as OR [95% CrI]). CrI, credible interval; CT, chemotherapy; HR, hazard ratio; niv, nivolumab; OR, odds ratio; ORR, objective response rate; pem, pembrolizumab.

All subgroup results were consistent with the base case for OS, PFS, and ORR. Tislelizumab + CT maintained the highest likelihood of being the top-ranked treatment in the PD-L1 ≥1 (TAP score ≥1% or CPS ≥1) and PD-L1 ≥5 (TAP score ≥5% or CPS ≥5) subgroups for OS and PFS (**Appendix D** in **Supplementary Material**), with a similar relative magnitude of treatment differences observed across the PD-L1 ≥1 and PD-L1 ≥5 subgroups. Similar results were also observed across the additional subgroup analyses for Asian regions versus ROW and platinum + fluoropyrimidine CT only. Forest plots of subgroup analyses for OS (**Figure 6**), PFS (**Figure 7**), and ORR (**Figure 8**) are presented below.

#### 3.2.2 Grade ≥3 TRAEs

**Figure 9:**
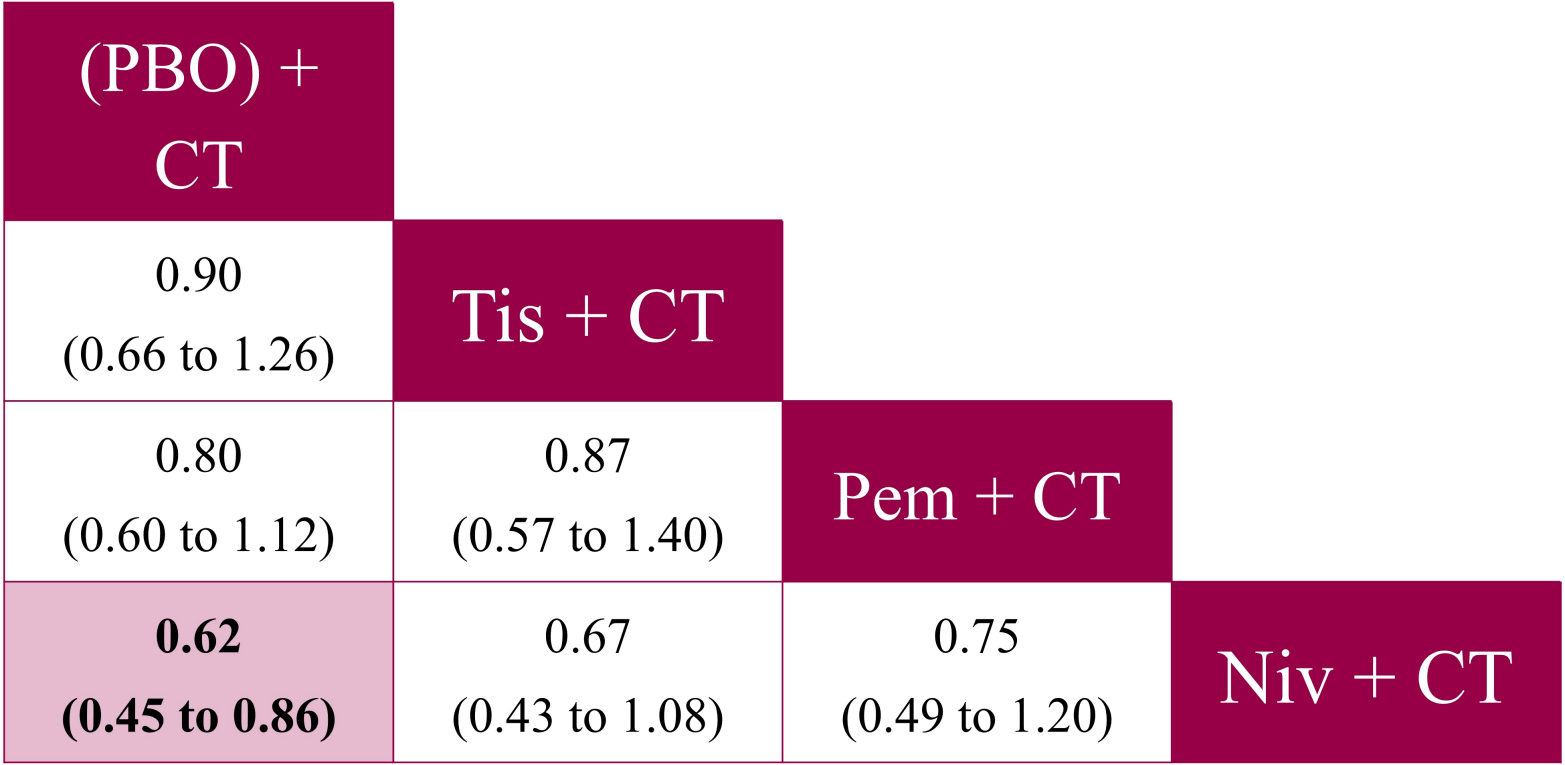
Pairwise comparisons from the fixed effects NMA for grade ≥3 TRAEs (reported as OR [95% CrI]) – ITT population analysis. CrI, credible interval; CT, chemotherapy; ITT, intent-to-treat; niv, nivolumab; NMA, network meta-analysis; OR, odds ratio; PBO, placebo; pem, pembrolizumab; tis, tislelizumab; TRAE, treatment-related adverse event.

Tislelizumab + CT was associated with similar odds of grade ≥3 TRAEs to pembrolizumab + CT (OR: 0.87, 95% CrI: 0.57 to 1.40) and nivolumab + CT (OR: 0.67, 95% CrI: 0.43 to 1.08), with no statistically significant differences observed (**Figure 9**). There were also no significant differences observed between pembrolizumab + CT and nivolumab + CT (OR: 0.75, 95% CrI: 0.49 to 1.20). Tislelizumab + CT was the highest-ranked IO regimen and a SUCRA score of 64% (P-best: 27%), indicating it had the lowest incidence odds of grade ≥3 TRAEs, relative to nivolumab + CT and pembrolizumab + CT (**Appendix Table 8** in **Supplementary Material**).

#### 3.2.3 Scenario analysis

The scenario analysis included additional evidence for nivolumab + ipilimumab, consisting of five treatment nodes informed by the same three trials as in the base case network (CheckMate 648, RATIONALE-306, and KEYNOTE-590) (**Appendix Figure 6** in **Supplementary Material**). The network consisted of 2,167 patients for OS, PFS, and ORR, and 2,321 patients for grade ≥3 TRAEs. Results from the scenario analysis were generally consistent with the base case (**Appendix E** in **Supplementary Material**), with the exception of nivolumab + ipilimumab being more favorable to nivolumab + CT for grade ≥3 TRAEs. Tislelizumab + CT performed similarly to all other IO regimens for OS, and was significantly more effective than nivolumab + CT and nivolumab + ipilimumab for PFS but similar to pembrolizumab + CT. In the ORR analyses, tislelizumab + CT was more effective than nivolumab + ipilimumab, and performed similarly to pembrolizumab + CT and nivolumab + CT.

## 4 Discussion

The addition of PD-1 inhibitors to CT represents a major therapeutic development for advanced or metastatic 1L ESCC, as treatment was previously only with CT regimens with limited survival benefits (41, 47). The introduction of anti–PD-1 agents like tislelizumab, nivolumab, and pembrolizumab further demonstrates the growing potential of targeting the PD-1/PD-L1 pathway. Finding the clinically optimum anti-PD-1 agent in this setting is critical to ensure appropriate access and clinical use. The computational power of NMA facilitates the ranking of several PD-1 inhibitors in terms of efficacy and safety to better inform clinical practice.

In this analysis, tislelizumab + CT was the highest ranked anti-PD-1 agent for OS, PFS, and grade ≥3 TRAE outcomes, and was significantly more favorable to nivolumab + CT for PFS. Collectively, these results highlight the favorable risk-benefit profile of tislelizumab + CT and provided a potential rationale for adopting the use of tislelizumab in ESCC.

In PD-L1 subgroup analyses, tislelizumab + CT was significantly more favorable to nivolumab + CT for PFS in patients with PD-L1 ≥1 (TAP score ≥1% or CPS ≥1) and PD-L1 ≥5 (TAP score ≥5% or CPS ≥5). Within both subgroups, tislelizumab + CT performed similarly to nivolumab + CT for OS and pembrolizumab + CT for OS and PFS. Although these results should be considered within the context of the differing PD-L1 expression scoring systems used across the included trials, a relatively high concordance between TAP score and CPS has been reported in terms of OPA (90% [95% CI: 86-93]) in the ESCC trial population (48). These results suggest that the relative efficacies of all three anti-PD-1 agents do not differ relative to the base case analyses based on patient PD-L1 level.

The subgroup analyses in patients receiving platinum + fluoropyrimidine as the CT backbone were consistent with the base case for OS, PFS, and ORR, demonstrating that tislelizumab performed similarly to both nivolumab and pembrolizumab in this patient subgroup. This suggests there may be flexibility in choosing the CT backbone based on individual patient factors and institutional or regional preferences. An additional scenario analysis was conducted to include nivolumab + ipilimumab, with similar results observed. Tislelizumab + CT was found to be comparable to nivolumab + ipilimumab in terms of OS and safety profile (grade ≥3 TRAEs) and was statistically more favorable to nivolumab + ipilimumab for PFS and ORR. Consistent results observed across both subgroup and scenario analyses lend additional confidence to these findings.

However, it should be noted that there were violations of the PH assumption in cross-trial comparisons involving nivolumab + ipilimumab, suggesting the treatment effect may not be constant over time and that the resulting HRs specifically against nivolumab + ipilimumab should be interpreted with caution. When considering within-trial data from RATIONALE-306, additional violations of the PH assumption were observed between tislelizumab + CT and placebo + CT specifically for OS. This violation has the potential to introduce time-dependent effects that may bias treatment estimates, particularly given that there are some differences between the three trials in follow-up time. Future models may consider alternative methods, such as fractional polynomial NMAs, restricted mean survival time analyses, or piecewise Cox PH models, to address these PH assumption violations (49, 50). However, it is not expected that these approaches would deviate substantially from the comparative results observed in this NMA. Further, such methods may be associated with overfitting and would require clinical input and validation of model choice.

Previous NMAs have also compared the efficacy of 1L IO therapies (35, 36), although certain relevant approved or novel regimens were not included for comparison. Ma et al. (36) conducted an NMA of six IO agents, including tislelizumab, pembrolizumab, and nivolumab, all in addition to CT. Of note, Ma et al. did not include nivolumab + ipilimumab (36), as was included in the additional scenario analysis of the current study. The present analysis reported that no significant differences were observed for OS between tislelizumab + CT, nivolumab + CT, and pembrolizumab + CT, similar to Ma et al. (36). Similarly, tislelizumab + CT ranked higher than both nivolumab + CT and pembrolizumab + CT for both OS and PFS in the analysis (36), as was seen in the current study. Moreover, in terms of safety profile, the ranking of tislelizumab + CT with respect to grade ≥3 TRAEs was generally consistent with findings from Ma et al. (36). The same NMA also ranked tislelizumab as having the third lowest rate of grade ≥3 TRAEs behind placebo + CT, while the current analysis ranked tislelizumab + CT as having the second lowest rate of grade ≥3 TRAEs behind placebo + CT (36).

More recently, Nian et al. conducted an NMA of nine IO therapies, including tislelizumab + CT, nivolumab + CT, nivolumab + ipilimumab, and pembrolizumab + CT (51). Similar to Ma et al. and the present analysis, there were no significant differences for OS between tislelizumab + CT, nivolumab + CT, nivolumab + ipilimumab, and pembrolizumab + CT (51, 52). Additionally, tislelizumab + CT was ranked higher than the same three comparator IO regimens for both OS and PFS, as observed in the current NMA and by Ma et al. (51, 52). Of note, the NMA by Nian et al. also found significantly more favorable PFS and ORR outcomes for tislelizumab + CT versus nivolumab + ipilimumab, with a similar magnitude of relative benefit (51).

Another recent NMA of eight IO agents was also conducted by Chen et al., including tislelizumab + CT, nivolumab + CT, nivolumab + ipilimumab, and pembrolizumab + CT (53). Both OS and PFS results were consistent with the present NMA. Similar to findings reported in Ma et al., Nian et al., and the current NMA’s scenario analysis, tislelizumab + CT was found statistically more favorable in terms of PFS than nivolumab + ipilimumab (51–53). The study’s safety findings in terms of grade ≥3 TRAEs were also consistent with those observed within the current analysis for tislelizumab + CT and other broadly approved IO therapies (53).

The present study has several strengths. First, analyses were performed according to best practice for conducting and reporting NMAs as described by the National Institute for Health and Care Excellence to ensure transparency and reproducibility (30). The NMAs were also informed by a recent, comprehensive SLR in adherence with best practices provided by PRISMA guidance. Further, all trials included in the SLR underwent a rigorous feasibility assessment to highlight any sources of inter-trial heterogeneity to ensure the validity of results. Key clinical subpopulations were also identified with clinical expert opinion for analysis to reflect the diversity of indications and reimbursement criteria for comparator treatments. To our knowledge, this is the first analysis to consider key PD-L1 expression subgroups aligned with approved indications for the use of tislelizumab + CT in 1L ESCC in the EU (PD-L1 TAP score ≥5%) and the US (PD-L1 ≥1) (13, 15). Further, the present NMA provides additional evidence confirming the assumption of equivalence of CT backbone regimens.

This study has the following limitations. First, network structures were sparse and connections between treatments were informed by a single trial, which increases the potential for biased treatment effect estimates, increases imprecision, and therefore reduces the robustness of results. Second, the limited number of trials informing each treatment comparison prevented the use of meta regression to adjust for potential sources of inter-trial heterogeneity. Additionally, indirect treatment comparisons, such as NMAs, rely on the assumption that the included trials are sufficiently similar, such that the effect estimate will not be biased by underlying differences in patient populations. In this analysis, minimal between-trial heterogeneity was observed, and potential differences were explored using subgroup analyses. As such, population-adjusted analyses were not conducted. Moreover, differences in CT backbones used across trials may have impacted these safety comparisons due to each CT regimen having distinct adverse event patterns (21, 39, 40).

Further, safety subgroup analyses were precluded by inconsistent reporting of safety outcomes in comparator trials (for example, only RATIONALE-306 defined adverse events of special interest, limiting cross-trial comparisons), which increases uncertainty of any findings in this study related to safety outcomes. Of note, KEYNOTE-590 only summarized safety outcomes for the broader esophageal cancer population, with adverse event data not being reported separately for the ESCC and esophageal adenocarcinoma subgroups. This may have limited comparisons of grade ≥3 TRAEs relative to pembrolizumab + CT, as RATIONALE-306 and CheckMate 648 specifically reported these data for the ESCC population. Safety comparisons may also be limited by the significant variation in treatment exposure and length of follow-up across the included trials; although, median treatment durations in the present NMA were similar across the included trials (5.7-7.7 months) (22, 39, 40). Treatments with longer exposure durations are more likely to exhibit a higher frequency of adverse events, potentially impacting comparisons in the current study of the cumulative incidence of grade ≥3 TRAEs.

In summary, the present study provides evidence on the relative efficacy and safety of relevant 1L IO regimens with broad regulatory approval for patients with unresectable, locally advanced or metastatic ESCC using NMAs. Probabilistically, tislelizumab was the top-ranked IO treatment across survival and safety analyses in the present NMA, although most pairwise comparisons were not statistically significant. Tislelizumab + CT was found to have a significant PFS benefit over nivolumab + CT, and similar efficacy to pembrolizumab + CT for OS and PFS. Tislelizumab + CT had a comparable frequency of grade ≥3 TRAEs to all treatments, including placebo + CT, whereas nivolumab + CT was associated with significantly increased incidence odds of grade ≥3 TRAEs relative to placebo + CT. Key subgroup analyses, including for PD-L1 expression (≥1% and ≥5% TAP score or CPS), Asia versus ROW, and underlying CT treatment, were generally consistent with the primary analysis, and showed comparable outcomes for tislelizumab + CT compared to other IO agents in combination with CT. Overall, based on results from the present analyses, tislelizumab + CT represents an effective treatment option for 1L ESCC.

## Data Availability

The original contributions presented in the study are included in the article/**Supplementary Material**. On request, and subject to certain criteria, conditions, and exceptions, BeOne Medicines, Ltd. will provide access to individual de-identified participant data from applicable BeOne Medicines-sponsored studies. BeOne Medicines shares data only when permitted by applicable data privacy and security laws and regulations, shares when it is feasible to do so without compromising the privacy of the study participants, and other considerations. Data requests may be submitted to ClinicalTrials@beonemed.com.
