# Supplementary material for: Tislelizumab efficacy and safety compared to other anti–PD-1s: a network meta-analysis of first-line therapies for unresectable, locally advanced or metastatic esophageal squamous cell carcinoma
Source: Front Immunol. 2026 Jan 6;16:1657085. doi: 10.3389/fimmu.2025.1657085 (PMC12816255; doi:10.3389/fimmu.2025.1657085)

Supplementary Material

1. Systematic Literature Review
   1. Search Strategy

**Date of the search:** 23 June 2023

**Database searched:**

- Ovid MEDLINE®
- Ovid MEDLINE Epub Ahead of Print, In-Process & Other Non-Indexed Citations and Daily
- Ovid Embase
- EBM Reviews - Cochrane Central Register of Controlled Trials
  - EBM Ovid EBM Reviews - Cochrane Database of Systematic Reviews

**Limits:**

- RCTs, SLRs & MAs Only
- Humans only
- Adults - 18 years and older
- Last 2 years of abstracts retained in Embase & CENTRAL
- Protocols & Opinion publications - removed

**Filters:**

**[Randomized Studies]**

(both MEDLINE and Embase filters have Phase 2-3 - additional terms to supplement RCTs filter) from:

Higgins JPT, Thomas J, Chandler J, Cumpston M, Li T, Page MJ, Welch VA (editors). *Cochrane Handbook for Systematic Reviews of Interventions* version 6.2 (updated February 2021). Cochrane, 2021. Available from www.training.cochrane.org/handbook.

**MEDLINE RCT Filter – Cochrane Handbook, 2019,** Box 3.c Cochrane Highly Sensitive Search Strategy for identifying randomized trials in MEDLINE: sensitivity-maximizing version (2008 revision); Ovid format

https://training.cochrane.org/handbook/current/chapter-04-technical-supplement-searching-and-selecting-studies#section-3-6-1

**Embase RCT sensitive Filter – Cochrane Handbook, 2019, Box 3.e** Cochrane Highly Sensitive Search Strategy for identifying controlled trials in Embase: (2018 revision) https://training.cochrane.org/handbook/current/chapter-04-technical-supplement-searching-and-selecting-studies#section-3-6-2

| **Search** - Clinical SLR | **Ovid Results** | **Results Deduplicated in Ovid** | **Results Deduplicated in EndNote** |
| --- | --- | --- | --- |
| ESCC & Tislelizumab & 12 Comparators & RCTs | 816 | 602 | 572 |

**MULTIFILE SEARCH**

Database(s): **EBM Reviews - Cochrane Central Register of Controlled Trials**May 2023**, EBM Reviews - Cochrane Database of Systematic Reviews**2005 to June 20, 2023**, Embase**1974 to 2023 June 22**, Ovid MEDLINE® and Epub Ahead of Print, In-Process, In-Data-Review & Other Non-Indexed Citations and Daily**1946 to June 22, 2023

**Search Strategy:**

| **#** | **Searches** | **Results** |
| --- | --- | --- |
| 1 | Esophageal Squamous Cell Carcinoma/ or (((esophag$ or oesophag$) adj5 (squamous$ or SC or adenosquamous$ or adeno-squamous$ or epidermoid$ or planocellular$ or prickle cell?) adj5 (neoplas$ or cancer$ or tumo?r$ or carcinoma$ or malignan$ or oncolog$ or adenocancer$ or adeno-cancer$ or adenoma$ or adenocarcinoma$ or adeno-carcinoma$ or blastoma$ or carcinosarcoma$ or carcino-sarcoma$ or adenoacanthoma$ or adeno-acanthoma$ or epithelioma$ or melanoma$ or mesenchymoma$ or sarcoma$ or thymoma$ or granuloma$ or choriocarcinoma$ or cancerogenes?s or carcinoid$)) or ((esophag$ or oesophag$) adj3 SCC) or (ESCC and (esophag$ or oesophag$))).ti,ab,kw,kf. [ESCC TERMS] | 48203 |
| 2 | exp Neoplasm Metastasis/ or Neoplasm Recurrence, Local/ or ((meta adj sta$) or metastas$ or metastatic$ or recur$ or secondar$ or relaps$ or advance$ or inoperab$ or disseminat$ or spread or migration or lethal$ or incurable or noncurable or non-curable or uncurable or progressive or terminal or invasive$ or aggressive$ or (late? adj2 stage$) or ((stage? or grade? or type?) adj2 (3a$ or 3b$ or 3c$ or III$ or 4a$ or 4b$ or IV or IVa or IVb or IVc)) or "stage 3" or "stage 4" or met or mets or N1 or N2? or N3? or pN1? or pN2? or pN3?).ti,ab,kw,kf. [METASTASIS] | 13763733 |
| 3 | 1 and 2 | 29095 |
| 4 | (tislelizumab$2 or tirelizumab$2 or bgb-a317 or bgba317 or bgn-1 or bgn1 or jhl-2108 or jhl2108 or vdt-482 or vdt482 or 1858168-59-8 or 0kvo411b3n).ti,ab,kw,kf,ot,hw,rn,nm. [TISLELIZUMAB TERMS] | 1731 |
| 5 | (atezolizumab$2 or anti-PDL1 or MPDL-3280A or MPDL3280A or RG-7446 or RG7446 or ro-5541267 or ro5541267 or Tecentriq$2 or Tecntriq$2 or 1380723-44-3 or 0INE2SFD9E or 52CMI0WC3Y).ti,ab,kw,kf,ot,hw,rn,nm. [ATEZOLIZUMAB TERMS] | 20778 |
| 6 | (avelumab$2 or bavencio$2 or msb-0010682 or msb-0010718c or msb0010682 or msb0010718c or msb-10682 or msb-10718c or msb10682 or msb10718c or pf-06834635 or pf-6834635 or pf06834635 or pf6834635 or KXG2PJ551I or 1537032-82-8).ti,ab,kw,kf,ot,hw,rn,nm. [AVELUMAB TERMS] | 7547 |
| 7 | (camrelizumab$2 or "anti-pd-1 monoclonal antibody" or shr-1210 or shr1210 or carilizumab$2 or carrelizumab$2 or 73096E137E or 1798286-48-2).ti,ab,kw,kf,ot,hw,rn,nm. [CAMRELIZUMAB TERMS] | 3604 |
| 8 | (durvalumab$2 or imfinzi$2 or medi-4736 or medi4736 or 28X28X9OKV or 1428935-60-7).ti,ab,kw,kf,ot,hw,rn,nm. [DURVALUMAB TERMS] | 12734 |
| 9 | Nivolumab/ or (nivolumab$2 or bms-936558 or bms-986213 or bms-986298 or cmab819 or bms936558 or bms986213 or bms986298 or cmab-819 or mdx-1106 or mdx1106 or ono-4538 or ono4538 or opdivo$2 or opdualag$2 or 31YO63LBSN or 946414-94-4).ti,ab,kw,kf,ot,hw,rn,nm. [NIVOLUMAB TERMS] | 50869 |
| 10 | (pembrolizumab$2 or keytruda$2 or lambrolizumab$2 or mk3475 or mk-1308a or mk-3475 or mk7684a or sch-900475 or sch900475 or "keylynk-010 component" or DPT0O3T46P or 1422183-02-5 or 1374853-91-4).ti,ab,kw,kf,ot,hw,rn,nm. [PEMBROLIZUMAB TERMS] | 49240 |
| 11 | (2072873-06-2 or 8fu7fq8upk or ibi308 or ibi-308 or sintilimab$2 or tyvyt$2 or who-10801).ti,ab,kw,kf,ot,hw,rn,nm. [SINTILIMAB TERMS] | 2269 |
| 12 | (1924598-82-2 or 8jxn261vva or js001 or js-001 or tab001 or tab-001 or teripalimab$2 or toripalimab$2 or treipril$2 or treprizumab$2 or tripleitriumab$2 or triprizumab$2 or tuoyi$2 or who-10820 or CHS-007).ti,ab,kw,kf,ot,hw,rn,nm. [TORIPALIMAB TERMS] | 1649 |
| 13 | (2231029-82-4 or hlx10 or hlx-10 or s3gqz2k36v or serplulimab$2).ti,ab,kw,kf,ot,hw,rn,nm. [SERPLULIMAB TERMS] | 101 |
| 14 | (2256084-03-2 or 90iqr2i6tr or cs1001 or cs-1001 or sugemalimab$2 or wbp315 or wbp-315 or wbp3155 or wbp-3155).ti,ab,kw,kf,ot,hw,rn,nm. [SUGEMALIMAB TERMS] | 148 |
| 15 | Ipilimumab/ or (ipilimumab$2 or bms-734016 or bms734016 or cs-1002 or cs1002 or ibi-310 or ibi310 or mdx-ctla-4 or mdx-010 or mdx-101 or mdx010 or mdx101 or strentarga$2 or yervoy$2 or 6T8C155666 or 477202-00-9).ti,ab,kw,kf,ot,hw,rn,nm. [IPILIMUMAB TERMS] | 32330 |
| 16 | (tremelimumab$2 or ticilimumab$2 or cp-675 or cp675 or cp675-cpd or cp-675 or cp-675-206 or cp-675206 or cp675206 or cp675-206 or pf-06753388 or QEN1X95CIX or 745013-59-6).ti,ab,kw,kf,ot,hw,rn,nm. [TREMELIMUMAB TERMS] | 5163 |
| 17 | Immune Checkpoint Inhibitors/ or ((Programmed Cell Death 1 Receptor/ or Programmed Cell Death 1 Ligand 2 Protein/) and (inhibit? or block?).ti,ab,kw,kf.) or ((immune$ adj3 checkpoint? adj3 (inhibit? or block?)) or (((programmed adj3 cell adj3 death) or PD-1 or PD-1-PD-L1 or PDCD1) adj3 (ligand? or inhibit? or block?)) or ((B7-H1 or B7H1 or "B7 homolog 1" or CD274 or CD273 or PDCD1LG1 or PDCD1LG2) adj3 (antigen? or protein?)) or ((Cytotoxic-T-Lymphocyte-Associated Protein-4 Inhibitor? or CTLA-4) adj3 (inhibit? or block?)) or ((ICI or ICIs) and "Immune Checkpoint") or BMS-1 or EX-A947 or HY-19991 or J-690233 or MFCD28978741 or s7911 or D000082082 or SCHEMBL16555159 or ZINC230477930 or 1675201-83-8).ti,ab,kw,kf,ot,hw,rn,nm. [IMMUNE CHECKPOINT PROTEINS TERMS] | 74124 |
| 18 | or/4-17 [INTERVENTIONS & COMPARATORS TERMS] | 157412 |
| 19 | (randomized controlled trial or controlled clinical trial).pt. or (randomized or placebo or randomly or trial or groups).ti,ab. or drug therapy.fs. [RCTs – MEDLINE sensitive Filter – Cochrane HSSS, 2019] | 15836332 |
| 20 | exp Randomized Controlled Trials as Topic/ or Clinical Trial, Phase II/ or Clinical Trial, Phase III/ or (equivalence trial or pragmatic clinical trial).pt. or (randomised or randomi#ation? or RCT or placebo$ or ((singl$ or doubl$ or trebl$ or tripl$) adj (mask$ or blind$ or dumm$)) or ((study or trial or CT) adj3 (phase 2 or phase 2a or phase 2b or phase 2c or phase II or phase IIa or phase IIb or phase IIc or phase 3 or phase 3a or phase 3b or phase 3c or phase III or phase IIIa or phase IIIb or phase IIIc or "phase? 2/3" or "phase? II/III" or "phase? 3/4" or "phase? III/IV")) or open label$).tw,kw,kf. [PHASE 2-3, OPEN LABEL - ADDITIONAL TERMS TO SUPPLEMENT RCTs FILTER] | 2541648 |
| 21 | 19 or 20 [RCTs ONLY] | 16128490 |
| 22 | (systematic review or systematic literature review or systematic scoping review or systematic narrative review or systematic qualitative review or systematic evidence review or systematic quantitative review or "systematic meta-review" or systematic critical review or systematic mixed studies review or systematic mapping review or systematic cochrane review or "systematic search and review" or systematic integrative review).ti. not comment.pt. not (protocol or protocols).ti. not MEDLINE.st. | 312794 |
| 23 | (1469-493X or 1361-6137).is. and review.pt. | 29334 |
| 24 | systematic review.pt. | 240370 |
| 25 | 22 or 23 or 24 [Ovid Expert Searches: SLR filter 2019] | 563697 |
| 26 | (meta-analy$ or metanaly$ or metaanaly$ or met-analy$).mp,pt. or review.pt. [SLR & MA - modified; Montori, 2004 - Balanced query, sn>sp Filter ] | 6796804 |
| 27 | Network Meta-Analysis/ or ((network adj1 (MA or MAs)) or (NMA or NMAs or MTC or MTCs or MAIC or MAICs or ITC or ITCs or STC or STCs) or indirect$ compar$ or (indirect treatment$ adj1 compar$) or (mixed treatment$ adj1 compar$) or (multiple treatment$ adj1 compar$) or (multi-treatment$ adj1 compar$) or simultaneous$ compar$ or mixed comparison?).tw,kw,kf. [Additional terms for MA, NMA, ITC] | 66206 |
| 28 | (cochrane or health technology assessment or evidence report or systematic reviews).jw. | 69774 |
| 29 | (systematic overview$ or evidence-based review$ or evidence-based overview$ or (evidence adj3 (review$ or overview$ or synthes$)) or meta-review$ or meta-overview$ or meta-synthes$ or metareview$ or metaoverview$ or metasynthes$ or rapid review$ or "review of reviews" or umbrella review? or technology assessment$ or HTA or HTAs).tw,kw,kf. [Additional terms for synonyms for systematic reviews and HTAs based on SLRs] | 230795 |
| 30 | or/25-29 [SLR & MA FILTERS - Combined] | 7030579 |
| 31 | 21 or 30 [RCTs & SLRs & MAs Filters] | 21280620 |
| 32 | 3 and 18 and 31 [mESCC & Drugs & Study Types TERMS] | 1200 |
| 33 | (Adolescent/ or exp Child/ or exp Infant/) not (exp Adult/ and (Adolescent/ or exp Child/ or exp Infant/)) [CHILDREN <19 REMOVE] | 4728322 |
| 34 | exp Animals/ not (exp Animals/ and Humans/) [ANIMAL STUDIES ONLY - REMOVE - MEDLINE] | 16826944 |
| 35 | (address or autobiography or bibliography or biography or comment or dictionary or directory or editorial or "expression of concern" or festschrift or historical article or interactive tutorial or lecture or legal case or legislation or news or newspaper article or patient education handout or personal narrative or portrait or video-audio media or webcast or (letter not (letter and randomized controlled trial))).pt. [Opinion publications - Remove -MEDLINE] | 4936789 |
| 36 | Clinical Trial Protocol.pt. | 571668 |
| 37 | 32 not (33 or 34 or 35 or 36) [CHILD <19, ANIMAL STUDIES, TRIAL PROTOCOLS and OPINION PUBLICATIONS - REMOVED - MEDLINE] | 1089 |
| 38 | 37 use ppez [MEDLINE results] | 237 |
| 39 | esophageal squamous cell carcinoma/ or (((esophag$ or oesophag$) adj5 (squamous$ or SC or adenosquamous$ or adeno-squamous$ or epidermoid$ or planocellular$ or prickle cell?) adj5 (neoplas$ or cancer$ or tumo?r$ or carcinoma$ or malignan$ or oncolog$ or adenocancer$ or adeno-cancer$ or adenoma$ or adenocarcinoma$ or adeno-carcinoma$ or blastoma$ or carcinosarcoma$ or carcino-sarcoma$ or adenoacanthoma$ or adeno-acanthoma$ or epithelioma$ or melanoma$ or mesenchymoma$ or sarcoma$ or thymoma$ or granuloma$ or choriocarcinoma$ or cancerogenes?s or carcinoid$)) or ((esophag$ or oesophag$) adj3 SCC) or (ESCC and (esophag$ or oesophag$))).ti,ab,kw,kf. [ESCC TERMS] | 48203 |
| 40 | exp metastasis/ or exp cancer recurrence/ or exp advanced cancer/ or ((meta adj sta$) or metastas$ or metastatic$ or recur$ or secondar$ or relaps$ or advance$ or inoperab$ or disseminat$ or spread or migration or lethal$ or incurable or noncurable or non-curable or uncurable or progressive or terminal or invasive$ or aggressive$ or (late? adj2 stage$) or ((stage? or grade? or type?) adj2 (3a$ or 3b$ or 3c$ or III$ or 4a$ or 4b$ or IV or IVa or IVb or IVc)) or "stage 3" or "stage 4" or met or mets or N1 or N2? or N3? or pN1? or pN2? or pN3?).ti,ab,kw,kf. [METASTASIS] | 13769518 |
| 41 | 39 and 40 | 29195 |
| 42 | tislelizumab/ or (tislelizumab$2 or tirelizumab$2 or bgb-a317 or bgba317 or bgn-1 or bgn1 or jhl-2108 or jhl2108 or vdt-482 or vdt482 or 1858168-59-8 or 0kvo411b3n).ti,ab,kw,kf,ot,rn,dq. [TISLELIZUMAB TERMS] | 1731 |
| 43 | atezolizumab/ or (atezolizumab$2 or anti-PDL1 or MPDL-3280A or MPDL3280A or RG-7446 or RG7446 or ro-5541267 or ro5541267 or Tecentriq$2 or Tecntriq$2 or 1380723-44-3 or 0INE2SFD9E or 52CMI0WC3Y).ti,ab,kw,kf,ot,rn,dq. [ATEZOLIZUMAB TERMS] | 20594 |
| 44 | avelumab/ or (avelumab$2 or bavencio$2 or msb-0010682 or msb-0010718c or msb0010682 or msb0010718c or msb-10682 or msb-10718c or msb10682 or msb10718c or pf-06834635 or pf-6834635 or pf06834635 or pf6834635 or KXG2PJ551I or 1537032-82-8).ti,ab,kw,kf,ot,rn,dq. [AVELUMAB TERMS] | 7543 |
| 45 | camrelizumab/ or (camrelizumab$2 or "anti-pd-1 monoclonal antibody" or shr-1210 or shr1210 or carilizumab$2 or carrelizumab$2 or 73096E137E or 1798286-48-2).ti,ab,kw,kf,ot,rn,dq. [CAMRELIZUMAB TERMS] | 3603 |
| 46 | durvalumab/ or (durvalumab$2 or imfinzi$2 or medi-4736 or medi4736 or 28X28X9OKV or 1428935-60-7).ti,ab,kw,kf,ot,rn,dq. [DURVALUMAB TERMS] | 12732 |
| 47 | nivolumab/ or (nivolumab$2 or bms-936558 or bms-986213 or bms-986298 or cmab819 or bms936558 or bms986213 or bms986298 or cmab-819 or mdx-1106 or mdx1106 or ono-4538 or ono4538 or opdivo$2 or opdualag$2 or 31YO63LBSN or 946414-94-4).ti,ab,kw,kf,ot,rn,dq. [NIVOLUMAB TERMS] | 50849 |
| 48 | pembrolizumab/ or (pembrolizumab$2 or keytruda$2 or lambrolizumab$2 or mk3475 or mk-1308a or mk-3475 or mk7684a or sch-900475 or sch900475 or "keylynk-010 component" or DPT0O3T46P or 1422183-02-5 or 1374853-91-4).ti,ab,kw,kf,ot,rn,dq. [PEMBROLIZUMAB TERMS] | 49222 |
| 49 | sintilimab/ or (2072873-06-2 or 8fu7fq8upk or ibi308 or ibi-308 or sintilimab$2 or tyvyt$2 or who-10801).ti,ab,kw,kf,ot,rn,dq. [SINTILIMAB TERMS] | 2269 |
| 50 | toripalimab/ or (1924598-82-2 or 8jxn261vva or js001 or js-001 or tab001 or tab-001 or teripalimab$2 or toripalimab$2 or treipril$2 or treprizumab$2 or tripleitriumab$2 or triprizumab$2 or tuoyi$2 or who-10820 or CHS-007).ti,ab,kw,kf,ot,rn,dq. [TORIPALIMAB TERMS] | 1649 |
| 51 | serplulimab/ or (2231029-82-4 or hlx10 or hlx-10 or s3gqz2k36v or serplulimab$2).ti,ab,kw,kf,ot,rn,dq. [SERPLULIMAB TERMS] | 101 |
| 52 | sugemalimab/ or (2256084-03-2 or 90iqr2i6tr or cs1001 or cs-1001 or sugemalimab$2 or wbp315 or wbp-315 or wbp3155 or wbp-3155).ti,ab,kw,kf,ot,rn,dq. [SUGEMALIMAB TERMS] | 148 |
| 53 | ipilimumab/ or (ipilimumab$2 or bms-734016 or bms734016 or cs-1002 or cs1002 or ibi-310 or ibi310 or mdx-ctla-4 or mdx-010 or mdx-101 or mdx010 or mdx101 or strentarga$2 or yervoy$2 or 6T8C155666 or 477202-00-9).ti,ab,kw,kf,ot,rn,dq. [IPILIMUMAB TERMS] | 32310 |
| 54 | tremelimumab/ or (tremelimumab$2 or ticilimumab$2 or cp-675 or cp675 or cp675-cpd or cp-675 or cp-675-206 or cp-675206 or cp675206 or cp675-206 or pf-06753388 or QEN1X95CIX or 745013-59-6).ti,ab,kw,kf,ot,rn,dq. [TREMELIMUMAB TERMS] | 5161 |
| 55 | immune checkpoint inhibitor/ or ((programmed death 1 receptor/ or programmed death 1 ligand 2/) and (inhibit? or block?).ti,ab,kw,kf.) or ((immune$ adj3 checkpoint? adj3 (inhibit? or block?)) or (((programmed adj3 cell adj3 death) or PD-1 or PD-1-PD-L1 or PDCD1) adj3 (ligand? or inhibit? or block?)) or ((B7-H1 or B7H1 or "B7 homolog 1" or CD274 or CD273 or PDCD1LG1 or PDCD1LG2) adj3 (antigen? or protein?)) or ((Cytotoxic-T-Lymphocyte-Associated Protein-4 Inhibitor? or CTLA-4) adj3 (inhibit? or block?)) or ((ICI or ICIs) and "Immune Checkpoint") or BMS-1 or EX-A947 or HY-19991 or J-690233 or MFCD28978741 or s7911 or D000082082 or SCHEMBL16555159 or ZINC230477930 or 1675201-83-8).ti,ab,kw,kf,ot,rn,dq. [IMMUNE CHECKPOINT PROTEINS TERMS] | 65484 |
| 56 | or/42-55 [INTERVENTIONS & COMPARATORS TERMS] | 150225 |
| 57 | Randomized controlled trial/ or Controlled clinical study/ or randomization/ or intermethod comparison/ or double blind procedure/ or human experiment/ or (compare or compared or comparison or trial).ti. or ((evaluated or evaluate or evaluating or assessed or assess) and (compare or compared or comparing or comparison)).ab. or (random$ or placebo or (open adj label) or ((double or single or doubly or singly) adj (blind or blinded or blindly)) or parallel group$1 or (crossover or cross over) or ((assign$ or match or matched or allocation) adj5 (alternate or group$1 or intervention$1 or patient$1 or subject$1 or participant$1)) or (assigned or allocated) or (controlled adj7 (study or design or trial)) or (volunteer or volunteers)).ti,ab. | 11789668 |
| 58 | (Cross-sectional study/ not (randomized controlled trial/ or controlled clinical study/ or controlled study/ or randomi?ed controlled.ti,ab. or control group$1.ti,ab.)) or ((((case adj control$) and random$) not randomi?ed controlled) or (nonrandom$ not random$) or "Random field$" or (random cluster adj3 sampl$)).ti,ab. or (Systematic review not (trial or study)).ti. or ((review.ab. and review.pt.) not trial.ti.) or ("we searched".ab. and (review.ti. or review.pt.)) or ("update review" or (databases adj4 searched)).ab. or ((rat or rats or mouse or mice or swine or porcine or murine or sheep or lambs or pigs or piglets or rabbit or rabbits or cat or cats or dog or dogs or cattle or bovine or monkey or monkeys or trout or marmoset$1).ti. and animal experiment/) or (Animal experiment/ not (human experiment/ or human/)) | 6169445 |
| 59 | 57 not 58 [RCTs – Embase sensitive Filter – Cochrane HSSS, 2019] | 10724925 |
| 60 | phase 2 clinical trial/ or phase 3 clinical trial/ or phase 4 clinical trial/ or (equivalence trial or pragmatic clinical trial).pt. or (randomised or randomi#ation? or RCT or placebo* or ((singl$ or doubl$ or trebl$ or tripl$) adj (mask$ or blind$ or dumm$)) or ((study or trial or CT) adj3 (phase 2 or phase 2a or phase 2b or phase 2c or phase II or phase IIa or phase IIb or phase IIc or phase 3 or phase 3a or phase 3b or phase 3c or phase III or phase IIIa or phase IIIb or phase IIIc or "phase? 2/3" or "phase? II/III" or "phase? 3/4" or "phase? III/IV")) or open label$).tw,kw,kf. [PHASE 2-4, OPEN LABEL - ADDITIONAL TERMS TO SUPPLEMENT RCTs FILTER] | 2244647 |
| 61 | 59 or 60 [RCTs ONLY] | 10985410 |
| 62 | exp meta analysis/ or ((meta adj analy$) or metaanalys$).mp. or (systematic adj (review? or overview?)).tw. or (cancerlit or cochrane or embase or psychlit or psyclit or psychinfo or psycinfo or cinahl or cinhal or science citation index or bids or reference lists or bibliograph$ or hand-search$ or manual search$ or relevant journals).ab. | 1278732 |
| 63 | (data extraction or selection criteria).ab. and review.pt. | 73445 |
| 64 | 62 or 63 [SLR & MA FILTER - Ovid Expert Searches: SLR filter 2019] | 1290450 |
| 65 | (meta-analy$ or metanaly$ or metaanaly$ or met-analy$).mp. or review.pt. [SLR & MA FILTER - modified and translated; Montori, 2004 - Balanced query, sn>sp Filter] | 6796804 |
| 66 | network meta-analysis/ or ((network adj1 (MA or MAs)) or (NMA or NMAs or MTC or MTCs or MAIC or MAICs or ITC or ITCs or STC or STCs) or indirect$ compar$ or (indirect treatment$ adj1 compar$) or (mixed treatment$ adj1 compar$) or (multiple treatment$ adj1 compar$) or (multi-treatment$ adj1 compar$) or simultaneous$ compar$ or mixed comparison?).tw,kw,kf. [Additional terms for MA, NMA, ITC] | 66206 |
| 67 | (cochrane or health technology assessment or evidence report or systematic reviews).jw. | 69774 |
| 68 | (systematic overview$ or evidence-based review$ or evidence-based overview$ or (evidence adj3 (review$ or overview$ or synthes$)) or meta-review$ or meta-overview$ or meta-synthes$ or metareview$ or metaoverview$ or metasynthes$ or rapid review$ or "review of reviews" or umbrella review? or technology assessment$ or HTA or HTAs).tw,kw,kf. [Additional terms for synonyms for systematic reviews and HTAs based on SLRs] | 230795 |
| 69 | or/64-68 [SLR & MA FILTERS - Combined] | 7166075 |
| 70 | 61 or 69 [RCTs & SLRs & MAs Filters] | 17568409 |
| 71 | 41 and 56 and 70 | 940 |
| 72 | (exp adolescent/ or exp child/ or exp fetus/) not (exp adult/ and (exp adolescent/ or exp child/ or exp fetus/)) [CHILDREN <18 REMOVE] | 4472111 |
| 73 | (exp animal/ or exp animal experimentation/ or exp animal model/ or exp animal experiment/ or nonhuman/ or exp vertebrate/) not (exp human/ or exp human experimentation/ or exp human experiment/) [ANIMAL STUDIES ONLY - REMOVE - EMBASE] | 12428674 |
| 74 | (editorial or note or short survey or tombstone).pt. or (letter.pt. not randomized controlled trial/) [OPINION PIECES REMOVE - Embase] | 5298209 |
| 75 | conference abstract.pt. [CONFERENCE ABSTRACTS] | 4798845 |
| 76 | 71 not (72 or 73 or 74) [CHILD <19, ANIMAL STUDIES and OPINION PUBLICATIONS - REMOVED - Embase] | 934 |
| 77 | 75 and 76 [CONFERENCE ABSTRACTS ONLY] | 198 |
| 78 | limit 77 to yr="2021 -Current" | 124 |
| 79 | 76 not 75 [CONFERENCE ABSTRACTS REMOVED] | 736 |
| 80 | 78 or 79 [LAST 2 YRS OF ABSTRACTS RETAINED - Embase] | 860 |
| 81 | 80 use oemezd [Embase results] | 506 |
| 82 | Esophageal Squamous Cell Carcinoma/ or (((esophag$ or oesophag$) adj5 (squamous$ or SC or adenosquamous$ or adeno-squamous$ or epidermoid$ or planocellular$ or prickle cell?) adj5 (neoplas$ or cancer$ or tumo?r$ or carcinoma$ or malignan$ or oncolog$ or adenocancer$ or adeno-cancer$ or adenoma$ or adenocarcinoma$ or adeno-carcinoma$ or blastoma$ or carcinosarcoma$ or carcino-sarcoma$ or adenoacanthoma$ or adeno-acanthoma$ or epithelioma$ or melanoma$ or mesenchymoma$ or sarcoma$ or thymoma$ or granuloma$ or choriocarcinoma$ or cancerogenes?s or carcinoid$)) or ((esophag$ or oesophag$) adj3 SCC) or (ESCC and (esophag$ or oesophag$))).ti,ab,kw. [ESCC TERMS] | 47895 |
| 83 | exp Neoplasm Metastasis/ or Neoplasm Recurrence, Local/ or ((meta adj sta$) or metastas$ or metastatic$ or recur$ or secondar$ or relaps$ or advance$ or inoperab$ or disseminat$ or spread or migration or lethal$ or incurable or noncurable or non-curable or uncurable or progressive or terminal or invasive$ or aggressive$ or (late? adj2 stage$) or ((stage? or grade? or type?) adj2 (3a$ or 3b$ or 3c$ or III$ or 4a$ or 4b$ or IV or IVa or IVb or IVc)) or "stage 3" or "stage 4" or met or mets or N1 or N2? or N3? or pN1? or pN2? or pN3?).ti,ab,kw. [METASTASIS] | 13723366 |
| 84 | 82 and 83 | 28930 |
| 85 | (tislelizumab$2 or tirelizumab$2 or bgb-a317 or bgba317 or bgn-1 or bgn1 or jhl-2108 or jhl2108 or vdt-482 or vdt482 or 1858168-59-8 or 0kvo411b3n).ti,ab,kw. [TISLELIZUMAB TERMS] | 985 |
| 86 | (atezolizumab$2 or anti-PDL1 or MPDL-3280A or MPDL3280A or RG-7446 or RG7446 or ro-5541267 or ro5541267 or Tecentriq$2 or Tecntriq$2 or 1380723-44-3 or 0INE2SFD9E or 52CMI0WC3Y).ti,ab,kw. [ATEZOLIZUMAB TERMS] | 11551 |
| 87 | (avelumab$2 or bavencio$2 or msb-0010682 or msb-0010718c or msb0010682 or msb0010718c or msb-10682 or msb-10718c or msb10682 or msb10718c or pf-06834635 or pf-6834635 or pf06834635 or pf6834635 or KXG2PJ551I or 1537032-82-8).ti,ab,kw. [AVELUMAB TERMS] | 3295 |
| 88 | (camrelizumab$2 or "anti-pd-1 monoclonal antibody" or shr-1210 or shr1210 or carilizumab$2 or carrelizumab$2 or 73096E137E or 1798286-48-2).ti,ab,kw. [CAMRELIZUMAB TERMS] | 2504 |
| 89 | (durvalumab$2 or imfinzi$2 or medi-4736 or medi4736 or 28X28X9OKV or 1428935-60-7).ti,ab,kw. [DURVALUMAB TERMS] | 6293 |
| 90 | Nivolumab/ or (nivolumab$2 or bms-936558 or bms-986213 or bms-986298 or cmab819 or bms936558 or bms986213 or bms986298 or cmab-819 or mdx-1106 or mdx1106 or ono-4538 or ono4538 or opdivo$2 or opdualag$2 or 31YO63LBSN or 946414-94-4).ti,ab,kw. [NIVOLUMAB TERMS] | 50689 |
| 91 | (pembrolizumab$2 or keytruda$2 or lambrolizumab$2 or mk3475 or mk-1308a or mk-3475 or mk7684a or sch-900475 or sch900475 or "keylynk-010 component" or DPT0O3T46P or 1422183-02-5 or 1374853-91-4).ti,ab,kw. [PEMBROLIZUMAB TERMS] | 30325 |
| 92 | (2072873-06-2 or 8fu7fq8upk or ibi308 or ibi-308 or sintilimab$2 or tyvyt$2 or who-10801).ti,ab,kw. [SINTILIMAB TERMS] | 1281 |
| 93 | (1924598-82-2 or 8jxn261vva or js001 or js-001 or tab001 or tab-001 or teripalimab$2 or toripalimab$2 or treipril$2 or treprizumab$2 or tripleitriumab$2 or triprizumab$2 or tuoyi$2 or who-10820 or CHS-007).ti,ab,kw. [TORIPALIMAB TERMS] | 853 |
| 94 | (2231029-82-4 or hlx10 or hlx-10 or s3gqz2k36v or serplulimab$2).ti,ab,kw. [SERPLULIMAB TERMS] | 71 |
| 95 | (2256084-03-2 or 90iqr2i6tr or cs1001 or cs-1001 or sugemalimab$2 or wbp315 or wbp-315 or wbp3155 or wbp-3155).ti,ab,kw. [SUGEMALIMAB TERMS] | 99 |
| 96 | Ipilimumab/ or (ipilimumab$2 or bms-734016 or bms734016 or cs-1002 or cs1002 or ibi-310 or ibi310 or mdx-ctla-4 or mdx-010 or mdx-101 or mdx010 or mdx101 or strentarga$2 or yervoy$2 or 6T8C155666 or 477202-00-9).ti,ab,kw. [IPILIMUMAB TERMS] | 32267 |
| 97 | (tremelimumab$2 or ticilimumab$2 or cp-675 or cp675 or cp675-cpd or cp-675 or cp-675-206 or cp-675206 or cp675206 or cp675-206 or pf-06753388 or QEN1X95CIX or 745013-59-6).ti,ab,kw. [TREMELIMUMAB TERMS] | 1974 |
| 98 | Immune Checkpoint Inhibitors/ or ((Programmed Cell Death 1 Receptor/ or Programmed Cell Death 1 Ligand 2 Protein/) and (inhibit? or block?).ti,ab,kw,kf.) or ((immune$ adj3 checkpoint? adj3 (inhibit? or block?)) or (((programmed adj3 cell adj3 death) or PD-1 or PD-1-PD-L1 or PDCD1) adj3 (ligand? or inhibit? or block?)) or ((B7-H1 or B7H1 or "B7 homolog 1" or CD274 or CD273 or PDCD1LG1 or PDCD1LG2) adj3 (antigen? or protein?)) or ((Cytotoxic-T-Lymphocyte-Associated Protein-4 Inhibitor? or CTLA-4) adj3 (inhibit? or block?)) or ((ICI or ICIs) and "Immune Checkpoint") or BMS-1 or EX-A947 or HY-19991 or J-690233 or MFCD28978741 or s7911 or D000082082 or SCHEMBL16555159 or ZINC230477930 or 1675201-83-8).ti,ab,kw. [IMMUNE CHECKPOINT PROTEINS TERMS] | 63965 |
| 99 | or/85-98 [INTERVENTIONS & COMPARATORS TERMS] | 141693 |
| 100 | 84 and 99 | 1329 |
| 101 | (Adolescent/ or exp Child/ or exp Infant/) not (exp Adult/ and (Adolescent/ or exp Child/ or exp Infant/)) [CHILDREN <19 REMOVE] | 4728322 |
| 102 | (editorial or note or comment or clinical trial protocol).pt. or (letter.pt. not randomized controlled trial/) [PROTOCOLS and OPINION PIECES REMOVE - CENTRAL] | 5792290 |
| 103 | 100 not (101 or 102) [PROTOCOLS and OPINION PIECES REMOVED - CENTRAL] | 1196 |
| 104 | Conference proceeding.pt. [CONFERENCE ABSTRACTS/PROCEEDINGS] | 221325 |
| 105 | 103 and 104 [CONFERENCE ABSTRACTS ONLY] | 76 |
| 106 | limit 105 to yr="2021 -Current" | 43 |
| 107 | 103 not 104 [CONFERENCE ABSTRACTS REMOVED] | 1120 |
| 108 | 106 or 107 [LAST 2 YRS OF ABSTRACTS RETAINED] | 1163 |
| 109 | 108 use cctr [CENTRAL results] | 73 |
| 110 | (((esophag$ or oesophag$) adj5 (squamous$ or SC or adenosquamous$ or adeno-squamous$ or epidermoid$ or planocellular$ or prickle cell?) adj5 (neoplas$ or cancer$ or tumo?r$ or carcinoma$ or malignan$ or oncolog$ or adenocancer$ or adeno-cancer$ or adenoma$ or adenocarcinoma$ or adeno-carcinoma$ or blastoma$ or carcinosarcoma$ or carcino-sarcoma$ or adenoacanthoma$ or adeno-acanthoma$ or epithelioma$ or melanoma$ or mesenchymoma$ or sarcoma$ or thymoma$ or granuloma$ or choriocarcinoma$ or cancerogenes?s or carcinoid$)) or ((esophag$ or oesophag$) adj3 SCC) or (ESCC and (esophag$ or oesophag$))).ti,ab,kw. [ESCC TERMS] | 42946 |
| 111 | ((meta adj sta$) or metastas$ or metastatic$ or recur$ or secondar$ or relaps$ or advance$ or inoperab$ or disseminat$ or spread or migration or lethal$ or incurable or noncurable or non-curable or uncurable or progressive or terminal or invasive$ or aggressive$ or (late? adj2 stage$) or ((stage? or grade? or type?) adj2 (3a$ or 3b$ or 3c$ or III$ or 4a$ or 4b$ or IV or IVa or IVb or IVc)) or "stage 3" or "stage 4" or met or mets or N1 or N2? or N3? or pN1? or pN2? or pN3?).ti,ab,kw. [METASTASIS] | 13540197 |
| 112 | 110 and 111 | 25371 |
| 113 | (tislelizumab$2 or tirelizumab$2 or bgb-a317 or bgba317 or bgn-1 or bgn1 or jhl-2108 or jhl2108 or vdt-482 or vdt482 or 1858168-59-8 or 0kvo411b3n).ti,ab,kw. [TISLELIZUMAB TERMS] | 985 |
| 114 | (atezolizumab$2 or anti-PDL1 or MPDL-3280A or MPDL3280A or RG-7446 or RG7446 or ro-5541267 or ro5541267 or Tecentriq$2 or Tecntriq$2 or 1380723-44-3 or 0INE2SFD9E or 52CMI0WC3Y).ti,ab,kw. [ATEZOLIZUMAB TERMS] | 11551 |
| 115 | (avelumab$2 or bavencio$2 or msb-0010682 or msb-0010718c or msb0010682 or msb0010718c or msb-10682 or msb-10718c or msb10682 or msb10718c or pf-06834635 or pf-6834635 or pf06834635 or pf6834635 or KXG2PJ551I or 1537032-82-8).ti,ab,kw. [AVELUMAB TERMS] | 3295 |
| 116 | (camrelizumab$2 or "anti-pd-1 monoclonal antibody" or shr-1210 or shr1210 or carilizumab$2 or carrelizumab$2 or 73096E137E or 1798286-48-2).ti,ab,kw. [CAMRELIZUMAB TERMS] | 2504 |
| 117 | (durvalumab$2 or imfinzi$2 or medi-4736 or medi4736 or 28X28X9OKV or 1428935-60-7).ti,ab,kw. [DURVALUMAB TERMS] | 6293 |
| 118 | (nivolumab$2 or bms-936558 or bms-986213 or bms-986298 or cmab819 or bms936558 or bms986213 or bms986298 or cmab-819 or mdx-1106 or mdx1106 or ono-4538 or ono4538 or opdivo$2 or opdualag$2 or 31YO63LBSN or 946414-94-4).ti,ab,kw. [NIVOLUMAB TERMS] | 31963 |
| 119 | (pembrolizumab$2 or keytruda$2 or lambrolizumab$2 or mk3475 or mk-1308a or mk-3475 or mk7684a or sch-900475 or sch900475 or "keylynk-010 component" or DPT0O3T46P or 1422183-02-5 or 1374853-91-4).ti,ab,kw. [PEMBROLIZUMAB TERMS] | 30325 |
| 120 | (2072873-06-2 or 8fu7fq8upk or ibi308 or ibi-308 or sintilimab$2 or tyvyt$2 or who-10801).ti,ab,kw. [SINTILIMAB TERMS] | 1281 |
| 121 | (1924598-82-2 or 8jxn261vva or js001 or js-001 or tab001 or tab-001 or teripalimab$2 or toripalimab$2 or treipril$2 or treprizumab$2 or tripleitriumab$2 or triprizumab$2 or tuoyi$2 or who-10820 or CHS-007).ti,ab,kw. [TORIPALIMAB TERMS] | 853 |
| 122 | (2231029-82-4 or hlx10 or hlx-10 or s3gqz2k36v or serplulimab$2).ti,ab,kw. [SERPLULIMAB TERMS] | 71 |
| 123 | (2256084-03-2 or 90iqr2i6tr or cs1001 or cs-1001 or sugemalimab$2 or wbp315 or wbp-315 or wbp3155 or wbp-3155).ti,ab,kw. [SUGEMALIMAB TERMS] | 99 |
| 124 | (ipilimumab$2 or bms-734016 or bms734016 or cs-1002 or cs1002 or ibi-310 or ibi310 or mdx-ctla-4 or mdx-010 or mdx-101 or mdx010 or mdx101 or strentarga$2 or yervoy$2 or 6T8C155666 or 477202-00-9).ti,ab,kw. [IPILIMUMAB TERMS] | 17771 |
| 125 | (tremelimumab$2 or ticilimumab$2 or cp-675 or cp675 or cp675-cpd or cp-675 or cp-675-206 or cp-675206 or cp675206 or cp675-206 or pf-06753388 or QEN1X95CIX or 745013-59-6).ti,ab,kw. [TREMELIMUMAB TERMS] | 1974 |
| 126 | ((immune$ adj3 checkpoint? adj3 (inhibit? or block?)) or (((programmed adj3 cell adj3 death) or PD-1 or PD-1-PD-L1 or PDCD1) adj3 (ligand? or inhibit? or block?)) or ((B7-H1 or B7H1 or "B7 homolog 1" or CD274 or CD273 or PDCD1LG1 or PDCD1LG2) adj3 (antigen? or protein?)) or ((Cytotoxic-T-Lymphocyte-Associated Protein-4 Inhibitor? or CTLA-4) adj3 (inhibit? or block?)) or ((ICI or ICIs) and "Immune Checkpoint") or BMS-1 or EX-A947 or HY-19991 or J-690233 or MFCD28978741 or s7911 or D000082082 or SCHEMBL16555159 or ZINC230477930 or 1675201-83-8).ti,ab,kw. [IMMUNE CHECKPOINT PROTEINS TERMS] | 41803 |
| 127 | or/113-126 [INTERVENTIONS & COMPARATORS TERMS] | 106569 |
| 128 | 112 and 127 | 1015 |
| 129 | 128 use coch [CDSR results] | 0 |
| 130 | 38 or 81 or 109 or 129 | 816 |
| **131** | **remove duplicates from 130** | **602** |

- 1. Supplemental Searches

Appendix Table 1: Selected databases and grey literature for supplemental searches

| **Search Engine** | **Database** |
| --- | --- |
| ***Ovid*** | - Ovid MEDLINE® - Ovid MEDLINE Epub Ahead of Print, In-Process & Other Non-Indexed Citations and Daily - Ovid Embase - EBM Reviews – Cochrane Central Register of Controlled Trials - EBM Ovid EBM Reviews – Cochrane Database of Systematic Reviews |
| ***Conferences^a^*** | - ASCO 2023 - ESMO-Asia 2021 - Blood 2021, 2022 - ISPOR 2021, 2022 - ISPOR EU 2021 - WCGI 2022, 2023 |
| ***Other Sources*** | - ClinicalTrials.gov https://www.clinicaltrials.gov/ - Australian New Zealand Clinical Trials Registry (ANZCTR) https://www.anzctr.org.au/ - International Clinical Trials Registry Platform (ICTRP) https://www.who.int/clinical-trials-registry-platform - Bibliographic search of select relevant SLRs - Korean Medical Article Database (KMbase) http://en.medric.or.kr/ - KoreaMed https://koreamed.org/ |
| ***HTA Agencies*** | - PBAC - HIRA - NICE |

^a^ The listed conferences are key conferences of interest; these conference years were not indexed in Embase and thus a hand search of the conference websites was performed

Abbreviations: ANZCTR, Australian New Zealand Clinical Trials Registry; ASCO, American Society of Clinical Oncology; ESMO, European Society for Medical Oncology; EBM, evidence-based medicine; HIRA, Health Insurance Review and Assessment Service; HTA, Health Technology Assessment; ICTRP, International Clinical Trials Registry Platform; ISPOR, International Society of Pharmacoeconomics and Outcomes Research; ISPOR EU, International Society of Pharmacoeconomics and Outcomes Research European Union; KMbase, Korean Medical Article Database; NICE, National Institute for Health and Care Excellence; PBAC, Pharmaceutical Benefits Advisory Committee; SLR, Systematic literature review; WCGI, World Congress on Gastrointestinal Cancer

- 1. Study Eligibility Criteria

Appendix Table 2: Study Selection Criteria

|  | **Inclusion Criteria** | **Exclusion Criteria** |
| --- | --- | --- |
| ***Population*** | - 1L unresectable, locally advanced, or metastatic ESCC - Adult patients (18+) | - 2L or later ESCC - Other cancers - Pediatric patients (<18 years) |
| ***Interventions*** | - Tislelizumab, nivolumab, pembrolizumab, camrelizumab, sintilimab, toripalimab, avelumab, durvalumab, atezolizumab, serplulimab, sugemalimab, tremelimumab, ipilimumab - Alone or in combination with chemotherapy/targeted therapy/other immunotherapy | - Those not listed |
| ***Comparators*** | - Any intervention above (alone or in combination with chemotherapy/targeted therapy/any other immunotherapy) - Chemotherapy - Placebo | - Those not listed |
| ***Outcomes*** | - OS, PFS, ORR, DoR, HRQoL, AE | - Any study not including at least one eligible outcome |
| ***Study Design*** | - Phase 2 and Phase 3 RCTs - Full text articles from database inception to present - Conference abstracts from last 2 years (2021, 2022, 2023) | - Phase 1 and 4 RCTs - Non-randomized studies - Conference abstracts prior to 2021 |
| ***Language*** | - English language articles | - Non-English articles |

Abbreviations: 1L, first-line; 2L, second line; AE, adverse events, CR, complete response; DoR, duration of response, ESCC, esophageal squamous cell carcinoma; HRQoL, health-related quality of life; ORR, objective response rate; OS, overall survival; PFS, progression-free survival; PR, partial response; RCT, randomized controlled trial

- 1. Assessment of Study Quality

Appendix Table 3: Assessment of Study Quality Results

| **Trial; NCT** | **Was randomization carried out appropriately?** | **Was the concealment of treatment allocation adequate?** | **Were the groups similar at the outset of the study in terms of prognostic factors?** | **Were the care providers, participants, and the outcome assessors blind to treatment allocation?** | **Were there any unexpected imbalances in drop-outs between groups?** | **Is there any evidence to suggest that the authors measured more outcomes than they reported?** | **Did the analysis include an ITT analysis? If so, was this appropriate and were appropriate methods used to account for missing data?** |
| --- | --- | --- | --- | --- | --- | --- | --- |
|  |  |  |  |  |  |  |  |
| RATIONALE-306; NCT03783442 | Yes | Yes | Yes | Yes | No | No | Not clear |
| CheckMate 648; NCT03143153 | Yes | Yes | Yes | No | No | No | Not clear |
| KEYNOTE-590; NCT03189719 | Yes | Yes | Yes | Yes | No | Not clear | Not clear |
| ASTRUM-007; NCT03958890 | Yes | Yes | Yes | Yes | No | No | No |
| JUPITER-06; NCT03829969 | Yes | Yes | Yes | Yes | No | No | No |
| ORIENT-15; NCT03748134 | Yes | Yes | Yes | Yes | No | No | Yes |
| ESCORT-1^st^; NCT03691090 | Yes | Yes | Yes | Yes | No | No | Not clear |

1. Summary of All Relevant Trials Identified in the SLR

Appendix Table 4: Summary of Relevant Trials Identified in the SLR

| **Trial Name; NCT** | **Data Cutoff Dates** | **Arms** (n patients) | **Median study follow-up time (months)** |
| --- | --- | --- | --- |
| RATIONALE-306 NCT03783442 | February 28, 2022^a^ | Tislelizumab plus chemotherapy  ITT (n = 326)  Safety (n = 324) | 16.3 |
|  |  | Placebo plus chemotherapy  ITT (n = 323)  Safety (n = 321) | 9.8 |
| CheckMate 648 NCT03143153 | January 18, 2021^a^ | Nivolumab plus chemotherapy  ITT (n = 321)  Safety (n = 310) | Minimum 13 months follow up |
|  |  | Nivolumab plus ipilimumab  ITT (n = 325)  Safety (n = 322) |  |
|  |  | Chemotherapy alone  ITT (n = 324)  Safety (n = 304) |  |
|  | NR (extended follow-up) | Nivolumab plus chemotherapy  ITT (n = 321)  Safety (n = NR) | Minimum 29 months follow up |
|  |  | Nivolumab plus ipilimumab  ITT (n = 325)  Safety (n = NR) |  |
|  |  | Chemotherapy alone  ITT (n = 324)  Safety (n = NR) |  |
| KEYNOTE-590  NCT03189719 | July 2, 2020^a^ | Pembrolizumab plus chemotherapy  ITT (n = 274)  Safety (n = 370) | 22.6 |
|  |  | Placebo plus chemotherapy  ITT (n = 274)  Safety (n = 370) |  |
|  | July 9, 2021 | Pembrolizumab plus chemotherapy  ITT (n = 274)  Safety (n = NR) | Additional 12 months of follow up |
|  |  | Placebo plus chemotherapy  ITT (n = 274)  Safety (n = NR) |  |
| ASTRUM-007  NCT03958890 | April 15, 2022^a^ | Serplulimab plus chemotherapy  ITT (n = 368)  Safety (n = 382) | 14.9 |
|  |  | Placebo plus chemotherapy  ITT (n = 183)  Safety (n = 168) | 15.0 |
| JUPITER-06  NCT03829969 | March 22, 2021^a^ | Toripalimab plus chemotherapy  ITT (n = 257)  Safety (n = 257) | 7.1 |
|  |  | Placebo plus chemotherapy  ITT (n = 257)  Safety (n = 257) | 7.1 |
| ORIENT-15  NCT03748134 | April 9, 2021 | Sintilimab plus chemotherapy  ITT (n = 327)  Safety (n = 327) | NR |
|  |  | Placebo plus chemotherapy  ITT (n = 332)  Safety (n = 332) |  |
|  | August 28, 2022^a^ | Sintilimab plus chemotherapy  ITT (n = 341)  Safety (n = NR) | 32.2 |
|  |  | Placebo plus chemotherapy  ITT (n = 349)  Safety (n = NR) |  |
| ESCORT-1^st^  NCT03691090 | October 30, 2020^a^ | Camrelizumab plus chemotherapy  ITT (n = 298)  Safety (n = 298) | 10.8 |
|  |  | Placebo plus chemotherapy  ITT (n = 298)  Safety (n = 297) |  |
| GEMSTONE-304  NCT04187352 | October 7, 2022^a^ | Sugemalimab plus chemotherapy  ITT (n = 358)  Safety (n = NR) | 15.2 |
|  |  | Placebo plus chemotherapy  ITT (n = 182)  Safety (n = NR) |  |

^a^ Data cutoff used in NMA analyses.

Abbreviations: HR, hazard ratio; NA, not applicable; NR, not reported; ORR, objective response rate; OS, overall survival; PFS, progression-free survival; TEAE, treatment-emergent adverse event; TRAE, treatment-related adverse event

1. SUCRA and P-Best Results

Appendix Table 5: Summary of SUCRA values from the fixed-effects NMA for OS

| **Treatment Arm** | **SUCRA (%)** | **Probability Best (%)** |
| --- | --- | --- |
| TIS + CT | 84 | 64 |
| PEM + CT | 61 | 22 |
| NIV + CT | 54 | 14 |
| (PBO) + CT | 0 | 0 |

Abbreviations: CT, chemotherapy; NIV, nivolumab; NMA, network meta-analysis; OS, overall survival; PBO, placebo; PEM, pembrolizumab; SUCRA, Surface Area Under the Cumulative Ranking Curve; TIS, tislelizumab.

Appendix Table 6: Summary of SUCRA values from the fixed-effects NMA for PFS

| **Treatment Arm** | **SUCRA (%)** | **Probability Best (%)** |
| --- | --- | --- |
| TIS + CT | 90 | 71 |
| PEM + CT | 74 | 28 |
| NIV + CT | 35 | 1 |
| (PBO) + CT | 1 | 0 |

Abbreviations: CT, chemotherapy; NIV, nivolumab; NMA, network meta-analysis; PBO, placebo; PEM, pembrolizumab; PFS, progression-free survival; SUCRA, Surface Area Under the Cumulative Ranking Curve; TIS, tislelizumab.

Appendix Table 7: Summary of SUCRA values from the fixed-effects NMA for ORR

| **Treatment Arm** | **SUCRA (%)** | **Probability Best (%)** |
| --- | --- | --- |
| NIV + CT | 87 | 65 |
| TIS + CT | 72 | 31 |
| PEM + CT | 41 | 4 |
| (PBO) + CT | 0 | 0 |

Abbreviations: CT, chemotherapy; NIV, nivolumab; NMA, network meta-analysis; PBO, placebo; PEM, pembrolizumab; PFS, progression-free survival; SUCRA, Surface Area Under the Cumulative Ranking Curve; TIS, tislelizumab.

Appendix Table 8: Summary of SUCRA values from the fixed-effects NMA for Grade ≥3 TRAE

| **Treatment Arm** | **SUCRA (%)** | **Probability Best (%)** |
| --- | --- | --- |
| (PBO) + CT | 87 | 65 |
| TIS + CT | 64 | 27 |
| PEM + CT | 43 | 8 |
| NIV + CT | 5 | 0 |

Abbreviations: CT, chemotherapy; NIV, nivolumab; NMA, network meta-analysis; PBO, placebo; PEM, pembrolizumab; SUCRA, Surface area Under the Cumulative Ranking curve; TIS, tislelizumab; TRAE, treatment-related adverse event.

1. Additional PD-L1 Positive Patient Subgroup Results

Appendix Figure 1: Fixed-effects League Table for OS in PD-L1 Positive Patients (TAP ≥ 1%, CPS ≥ 1%)

| TIS + CT |  |  |  |
| --- | --- | --- | --- |
| 0.93  (0.68 to 1.26) | PEM + CT |  |  |
| 0.93  (0.69 to 1.25) | 1.00  (0.75 to 1.34) | NIV + CT |  |
| **0.64**  **(0.51 to 0.80)** | **0.69**  **(0.56 to 0.85)** | **0.69**  **(0.56 to 0.85)** | (PBO) + CT |

Note: Subgroup analysis; reported as HR [95% CrI], HR < 1 implies that column is better than row. Bolded text represents statistically significant results. The treatment with the most favorable estimate is positioned at the top left corner; the second, third, and fourth most favorable treatments are shown in descending order to the lower right.

Appendix Table 9: Summary of SUCRA values from the fixed-effects NMA for OS in PD-L1 Positive Patients (TAP ≥ 1%, CPS ≥ 1%)

| **Treatment Arm** | **SUCRA (%)** | **Probability Best (%)** |
| --- | --- | --- |
| TIS + CT | 79 | 55 |
| PEM + CT | 61 | 23 |
| NIV + CT | 60 | 22 |
| (PBO) + CT | 0 | 0 |

Appendix Figure 2: Fixed-effects League Table for OS in PD-L1 Positive Patients (TAP ≥ 5%, CPS ≥ 5%)

| TIS + CT |  |  |  |
| --- | --- | --- | --- |
| 0.88  (0.61 to 1.26) | PEM + CT |  |  |
| 0.83  (0.58 to 1.17) | 0.94  (0.67 to 1.31) | NIV + CT |  |
| **0.57**  **(0.44 to 0.74)** | **0.65**  **(0.51 to 0.83)** | **0.69**  **(0.55 to 0.87)** | (PBO) + CT |

Note: Subgroup analysis; reported as HR [95% CrI], HR < 1 implies that column is better than row. Bolded text represents statistically significant results. The treatment with the most favorable estimate is positioned at the top left corner; the second, third, and fourth most favorable treatments are shown in descending order to the lower right.

Appendix Table 10: Summary of SUCRA values from the fixed-effects NMA for OS in PD-L1 Positive Patients (TAP ≥ 5%, CPS ≥ 5%)

| **Treatment Arm** | **SUCRA (%)** | **Probability Best (%)** |
| --- | --- | --- |
| TIS + CT | 87 | 70 |
| PEM + CT | 62 | 21 |
| NIV + CT | 50 | 9 |
| (PBO) + CT | 0 | 0 |

Appendix Figure 3: Fixed-effects League Table for PFS in PD-L1 Positive Patients (TAP ≥ 1%, CPS ≥ 1%)

| TIS + CT |  |  |  |
| --- | --- | --- | --- |
| 0.87  (0.64 to 1.19) | PEM + CT |  |  |
| **0.72**  **(0.53 to 0.97)** | 0.82  (0.61 to 1.10) | NIV + CT |  |
| **0.56**  **(0.45 to 0.70)** | **0.64**  **(0.52 to 0.79)** | **0.78**  **(0.64 to 0.95)** | (PBO) + CT |

Note: Subgroup analysis; reported as HR [95% CrI], HR < 1 implies that column is better than row. Bolded text represents statistically significant results. The treatment with the most favorable estimate is positioned at the top left corner; the second, third, and fourth most favorable treatments are shown in descending order to the lower right.

Appendix Table 11: Summary of SUCRA values from the fixed-effects NMA for PFS in PD-L1 Positive Patients (TAP ≥ 1%, CPS ≥ 1%)

| **Treatment Arm** | **SUCRA (%)** | **Probability Best (%)** |
| --- | --- | --- |
| TIS + CT | 93 | 80 |
| PEM + CT | 70 | 19 |
| NIV + CT | 37 | 1 |
| (PBO) + CT | 0 | 0 |

Appendix Figure 4: Fixed-effects League Table for PFS in PD-L1 Positive Patients (TAP ≥ 5%, CPS ≥ 5%)

| TIS + CT |  |  |  |
| --- | --- | --- | --- |
| 0.91  (0.69 to 1.21) | PEM + CT |  |  |
| **0.64**  **(0.49 to 0.84)** | **0.70**  **(0.50 to 0.99)** | NIV + CT |  |
| **0.52**  **(0.46 to 0.59)** | **0.57**  **(0.44 to 0.73)** | 0.81  (0.64 to 1.02) | (PBO) + CT |

Note: Subgroup analysis; reported as HR [95% CrI], HR < 1 implies that column is better than row. Bolded text represents statistically significant results. The treatment with the most favorable estimate is positioned at the top left corner; the second, third, and fourth most favorable treatments are shown in descending order to the lower right.

Appendix Table 12: Summary of SUCRA values from the fixed-effects NMA for PFS in PD-L1 Positive Patients (TAP ≥ 5%, CPS ≥ 5%)

| **Treatment Arm** | **SUCRA (%)** | **Probability Best (%)** |
| --- | --- | --- |
| TIS + CT | 91 | 74 |
| PEM + CT | 75 | 26 |
| NIV + CT | 33 | 0 |
| (PBO) + CT | 1 | 0 |

1. Scenario Analysis Results

Appendix Figure 5: Evidence network for all outcomes in the Scenario Analysis


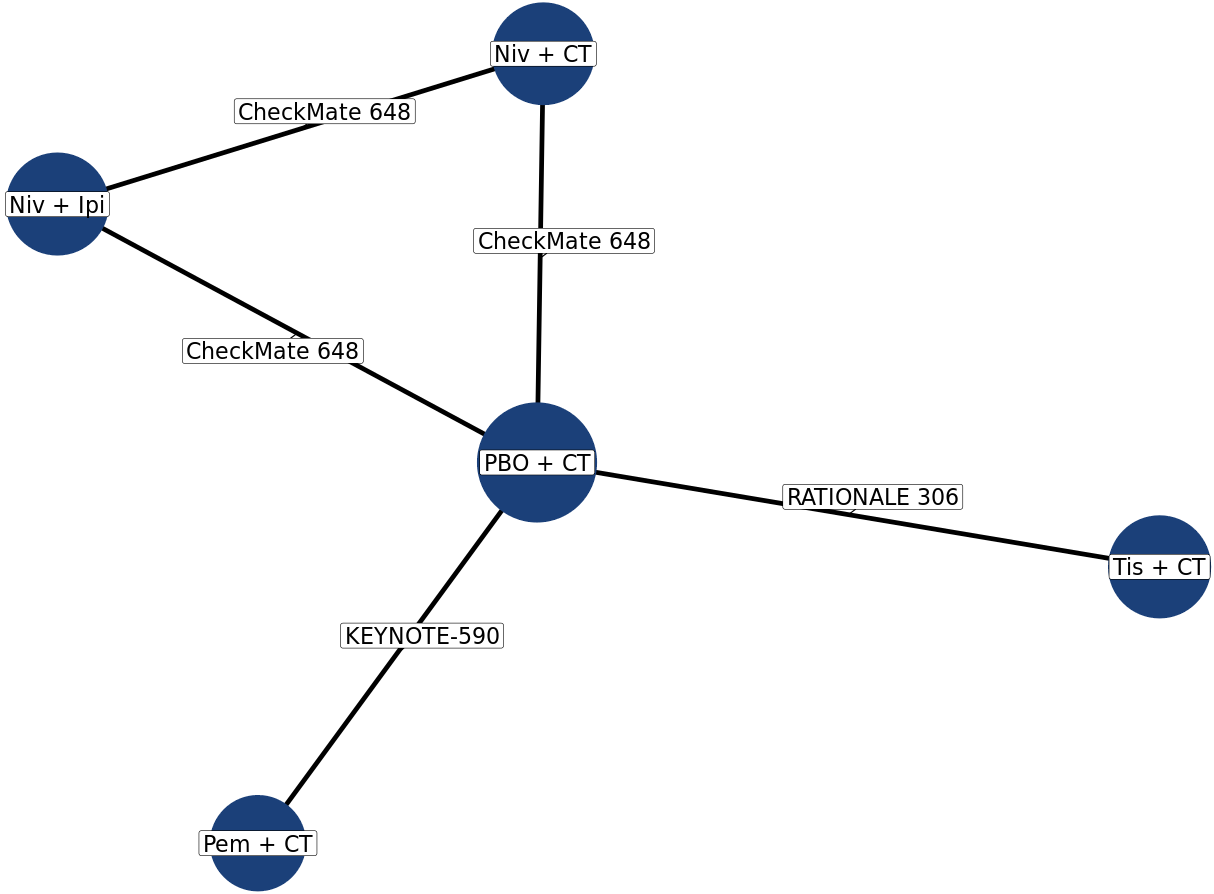


Appendix Figure 6: Fixed-effects League Table for OS in the Scenario Analysis (reported as HR [95% CrI])

| TIS + CT |  |  |  |  |
| --- | --- | --- | --- | --- |
| 0.92  (0.70 to 1.21) | PEM + CT |  |  |  |
| 0.89  (0.68 to 1.17) | 0.97  (0.74 to 1.27) | NIV + CT |  |  |
| 0.85  (0.64 to 1.11) | 0.92  (0.71 to 1.21) | 0.95  (0.79 to 1.15) | NIV + IPI |  |
| **0.66**  **(0.54 to 0.80)** | **0.72**  **(0.59 to 0.87)** | **0.74**  **(0.61 to 0.89)** | **0.78**  **(0.64 to 0.94)** | (PBO) + CT |

Note: Scenario analysis; reported as HR [95% CrI], HR < 1 implies that column is better than row. Bolded text represents statistically significant results. The treatment with the most favorable estimate is positioned at the top left corner; the second, third, fourth, and fifth most favorable treatments are shown in descending order to the lower right.

Appendix Table 13: Summary of SUCRA values from the fixed-effects NMA for OS in the Scenario Analysis

| **Treatment Arm** | **SUCRA (%)** | **Probability Best (%)** |
| --- | --- | --- |
| TIS + CT | 85 | 62 |
| PEM + CT | 64 | 21 |
| NIV + CT | 58 | 13 |
| NIV + IPI | 42 | 4 |
| (PBO) + CT | 0 | 0 |

Appendix Figure 7: Fixed-effects League Table for PFS in the Scenario Analysis (reported as HR [95% CrI])

| TIS + CT |  |  |  |  |
| --- | --- | --- | --- | --- |
| 0.92 (0.70 to 1.22) | PEM + CT |  |  |  |
| **0.74 (0.56 to 0.99)** | 0.80 (0.61 to 1.05) | NIV + CT |  |  |
| **0.60 (0.49 to 0.74)** | **0.65 (0.54 to 0.78)** | **0.81** **(0.67 to 0.98)** | (PBO) + CT |  |
| **0.48**  **(0.36 to 0.63)** | **0.52**  **(0.40 to 0.67)** | **0.64**  **(0.53 to 0.78)** | **0.79**  **(0.66 to 0.96)** | NIV + IPI |

Note: Scenario analysis; Reported as HR [95% CrI], HR < 1 implies that column is better than row. Bolded text represents statistically significant results. The treatment with the most favorable estimate is positioned at the top left corner; the second, third, fourth, and fifth most favorable treatments are shown in descending order to the lower right.

Appendix Table 14: Summary of SUCRA values from the fixed-effects NMA for PFS in the Scenario Analysis

| **Treatment Arm** | **SUCRA (%)** | **Probability Best (%)** |
| --- | --- | --- |
| TIS + CT | 92 | 71 |
| PEM + CT | 81 | 28 |
| NIV + CT | 51 | 1 |
| (PBO) + CT | 25 | 0 |
| NIV + IPI | 0 | 0 |

Appendix Figure 8: Fixed-effects League Table for ORR in the Scenario Analysis (reported as OR [95% CrI])

| NIV + CT |  |  |  |  |
| --- | --- | --- | --- | --- |
| 1.12  (0.70 to 1.76) | TIS + CT |  |  |  |
| 1.42  (0.88 to 2.30) | 1.27  (0.79 to 2.05) | PEM + CT |  |  |
| **2.36**  **(1.70** **to 3.27)** | **2.11**  **(1.32 to 3.40)** | **1.66**  **(1.02 to 2.72)** | NIV + IPI |  |
| **2.46**  **(1.78 to 3.34)** | **2.21**  **(1.61 to 3.04)** | **1.73**  **(1.22 to 2.46)** | 1.04  (0.74 to 1.47) | (PBO) + CT |

Note: Scenario analysis; Reported as OR [95% CrI], OR > 1 implies that column is better than row. Bolded text represents statistically significant results. The treatment with the most favorable estimate is positioned at the top left corner; the second, third, fourth, and fifth most favorable treatments are shown in descending order to the lower right.

Appendix Table 15: Summary of SUCRA values from the fixed-effects NMA for ORR in the Scenario Analysis

| **Treatment Arm** | **SUCRA (%)** | **Probability Best (%)** |
| --- | --- | --- |
| NIV + CT | 90 | 65 |
| TIS + CT | 79 | 31 |
| PEM + CT | 55 | 4 |
| NIV + IPI | 15 | 0 |
| (PBO) + CT | 10 | 0 |

Appendix Table 16: Summary of Subgroup Analyses for OS, PFS, and ORR in the Scenario Analysis

|  | **OS** | | | **PFS** | | | **ORR** | | |
| --- | --- | --- | --- | --- | --- | --- | --- | --- | --- |
|  | **TIS+CT vs. NIV+CT**  **HR (95% CrI)** | **TIS+CT vs. PEM+CT**  **HR (95% CrI)** | **TIS+CT vs. NIV+IPI**  **HR (95% CrI)** | **TIS+CT vs. NIV+CT**  **HR (95% CrI)** | **TIS+CT vs. PEM+CT**  **HR (95% CrI)** | **TIS+CT vs. NIV+IPI**  **HR (95% CrI)** | **TIS+CT vs. NIV+CT**  **OR (95% CrI)** | **TIS+CT vs. PEM+CT**  **OR (95% CrI)** | **TIS+CT vs. NIV+IPI**  **OR (95% CrI)** |
| PD-L1 ≥1% (TAP ≥1%, CPS ≥1) | 0.93  (0.68 to 1.26) | N/A | 0.84  (0.62 to 1.14) | N/A | N/A | N/A | N/A | N/A | N/A |
| PD-L1 ≥5% (TAP ≥5%, CPS ≥5) | 0.82  (0.58 to 1.18) | N/A | 0.79  (0.56 to 1.13) | N/A | N/A | N/A | N/A | N/A | N/A |
| Platinum plus Fluoropyrimidine only | 0.88  (0.62 to 1.25) | 0.90  (0.64 to 1.29) | 0.84  (0.59 to 1.18) | 0.82  (0.59 to 1.14) | 1.02 (0.74 to 1.41) | **0.52**  **(0.38 to 0.73)** | 0.98 (0.55 to 1.75) | 1.39  (0.77 to 2.52) | **2.31**  **(1.29 to 4.19)** |
| Asia Regions | 0.91  (0.63 to 1.33) | 0.96  (0.58 to 1.59) | 0.98  (0.68 to 1.42) | 0.82  (0.57 to 1.19) | 1.10 (0.69 to 1.72) | **0.54**  **(0.37 to 0.78)** | 0.59 (0.31 to 1.11) | 1.26 (0.56 to 2.80) | 1.36 (0.71 to 2.59) |
| ROW Regions | 0.86  (0.52 to 1.42) | 0.77  (0.49 to 1.20) | 0.92  (0.56 to 1.53) | N/A | N/A | N/A | N/A | N/A | N/A |

Note: HR < 1 or OR > 1 implies that tislelizumab + CT is more favorable than the comparator. Bolded text represents statistically significant results. Measurement system varied by trial: Tis + CT data are reflective of the TAP method, Pem + CT data are reflective of the CPS method, Niv + CT data are reflective of the TPS method.

Appendix Figure 9: Fixed-effects League Table for Grade ≥3 TRAE in the Scenario Analysis (reported as OR [95% CrI])

| NIV + IPI |  |  |  |  |
| --- | --- | --- | --- | --- |
| 0.86  (0.62 to 1.20) | (PBO) + CT |  |  |  |
| 0.78  (0.48 to 1.25) | 0.91  (0.65 to 1.26) | TIS + CT |  |  |
| 0.71  (0.45 to 1.11) | 0.82  (0.60 to 1.12) | 0.90  (0.57 to 1.42) | PEM + CT |  |
| **0.54**  **(0.39 to 0.74)** | **0.62**  **(0.45 to 0.86)** | 0.69  (0.44 to 1.09) | 0.76  (0.49 to 1.20) | NIV + CT |

Note: Scenario analysis; Reported as OR [95% CrI], OR < 1 implies that column is better than row. Bolded text represents statistically significant results. The treatment with the most favorable estimate is positioned at the top left corner; the second, third, fourth, and fifth most favorable treatments are shown in descending order to the lower right.

Appendix Table 17: Summary of SUCRA values from the fixed-effects NMA for Grade ≥3 TRAE in the Scenario Analysis

| **Treatment Arm** | **SUCRA (%)** | **Probability Best (%)** |
| --- | --- | --- |
| NIV + IPI | 90 | 74 |
| (PBO) + CT | 70 | 12 |
| TIS + CT | 51 | 11 |
| PEM + CT | 34 | 3 |
| NIV + CT | 4 | 0 |

1. Cross-Trial Proportional Hazards Assumption Results

Appendix Figure 10: Cumulative hazard log plots for OS, ITT population (RATIONALE-306, CheckMate 648, and KEYNOTE-590)


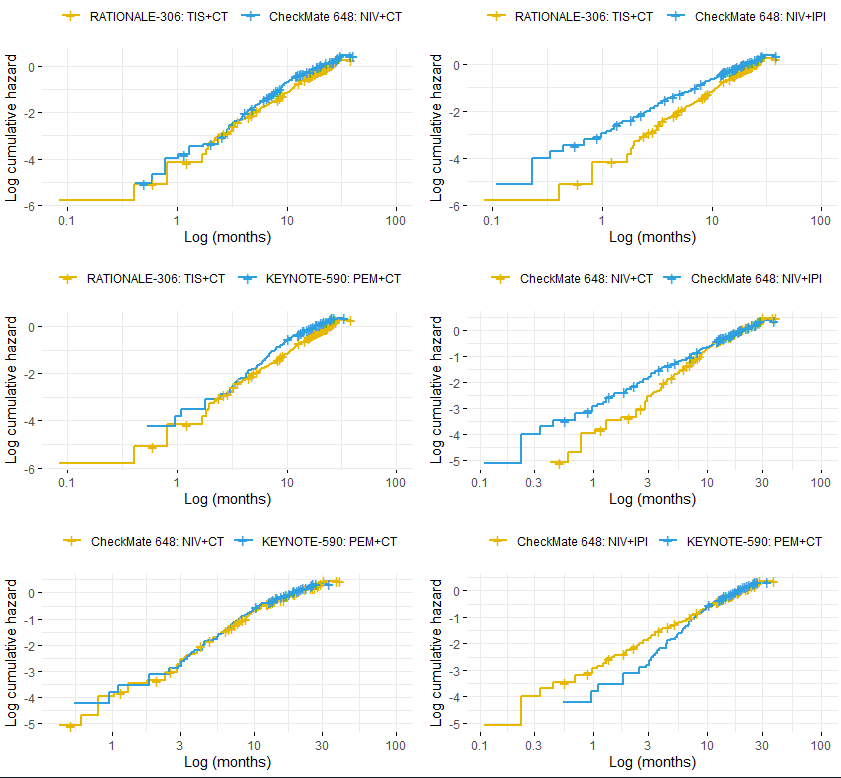


Appendix Figure 11: Schoenfeld residual plots with Grambsch–Therneau p-values for OS, ITT population (RATIONALE-306, CheckMate 648, and KEYNOTE-590)


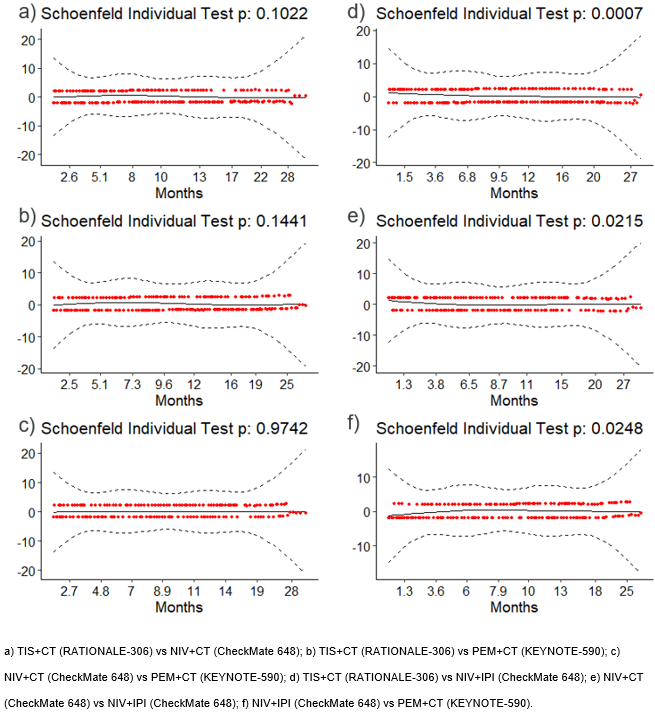


a) TIS + CT (RATIONALE-306) vs. NIV + CT (CheckMate 648); b) TIS + CT (RATIONALE-306) vs. PEM + CT (KEYNOTE-590); c) NIV + CT (CheckMate 648) vs. PEM + CT (KEYNOTE-590); d) TIS + CT (RATIONALE-306) vs. NIV + IPI (CheckMate 648); e) NIV + CT (CheckMate 648) vs. NIV + IPI (CheckMate 648); f) NIV + IPI (CheckMate 648) vs. PEM + CT (KEYNOTE-590).

Appendix Figure 12: Cumulative hazard log plots for PFS, ITT population (RATIONALE-306, CheckMate 648, and KEYNOTE-590)


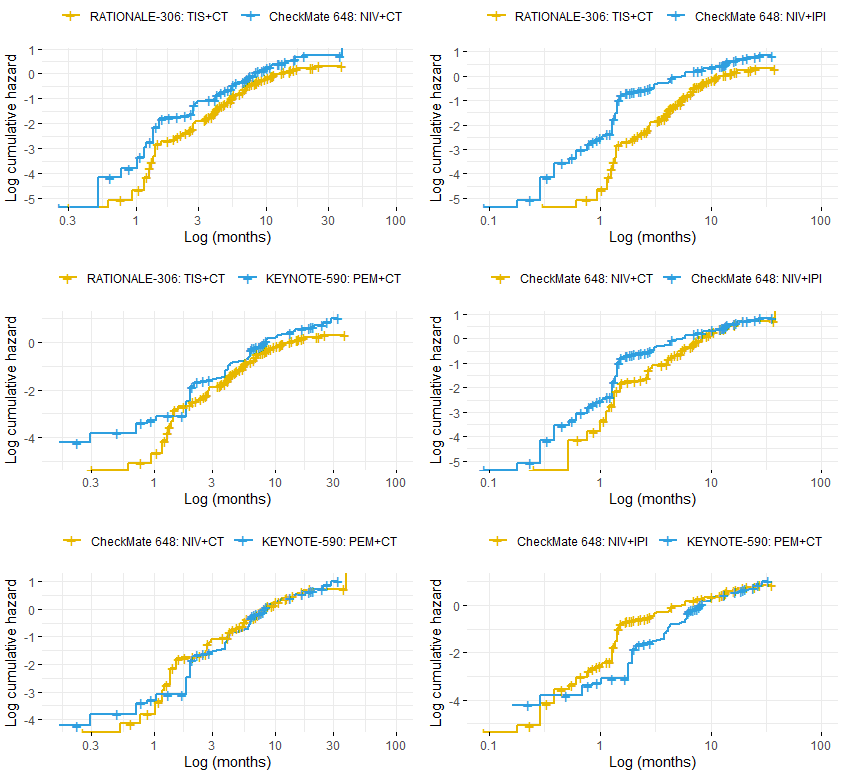


Appendix Figure 13: Schoenfeld residual plots with Grambsch–Therneau p-values for PFS, ITT population (RATIONALE-306, CheckMate 648, and KEYNOTE-590)


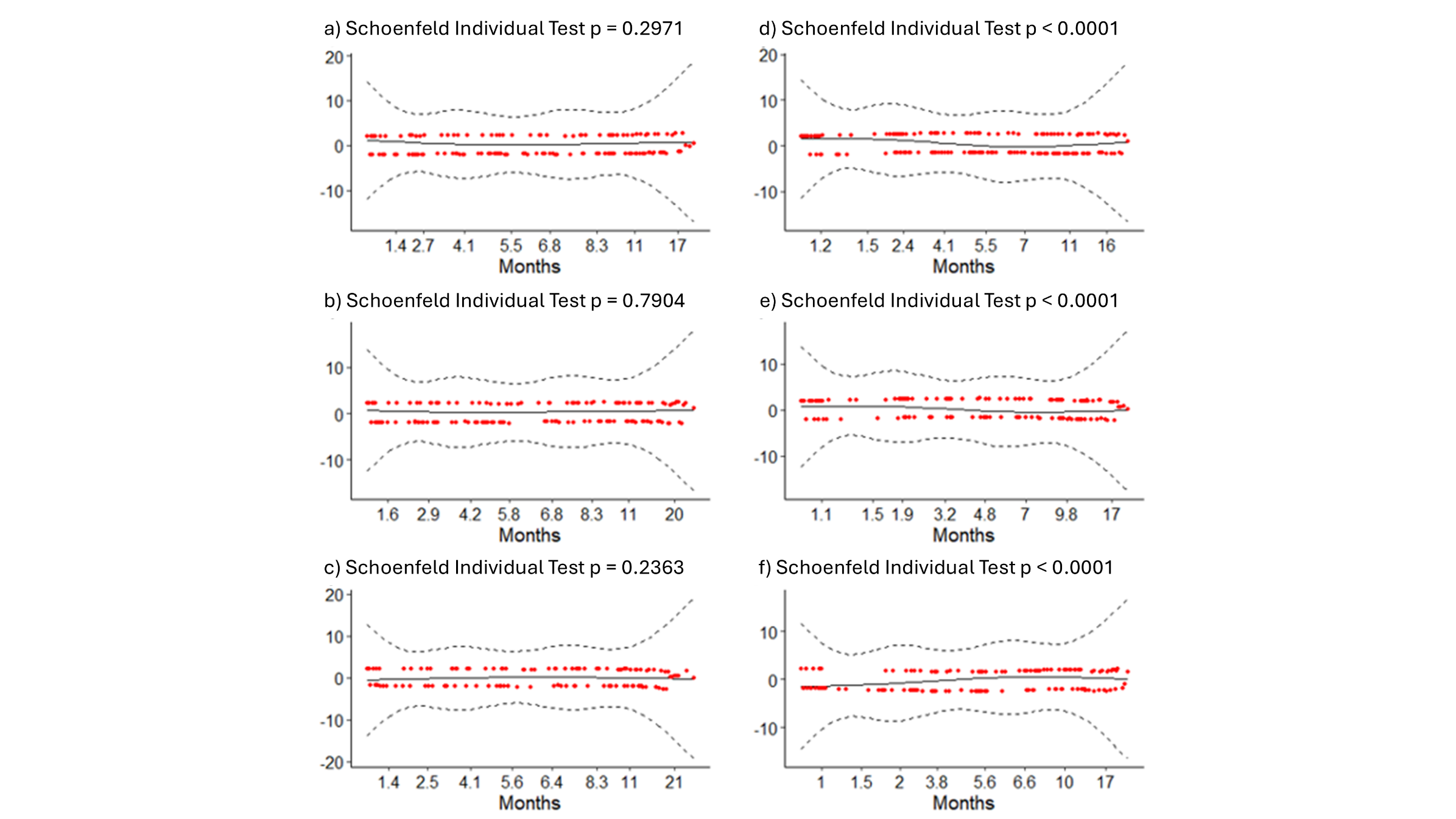


a) TIS + CT (RATIONALE-306) vs. NIV + CT (CheckMate 648); b) TIS + CT (RATIONALE-306) vs. PEM + CT (KEYNOTE-590); c) NIV + CT (CheckMate 648) vs. PEM + CT (KEYNOTE-590); d) TIS + CT (RATIONALE-306) vs. NIV + IPI (CheckMate 648); e) NIV + CT (CheckMate 648) vs. NIV + IPI (CheckMate 648); f) NIV + IPI (CheckMate 648) vs. PEM + CT (KEYNOTE-590).

1. Within-Trial Proportional Hazards Assumption Results

Appendix Figure 14: Cumulative hazard log plots for OS, ITT population (RATIONALE-306)


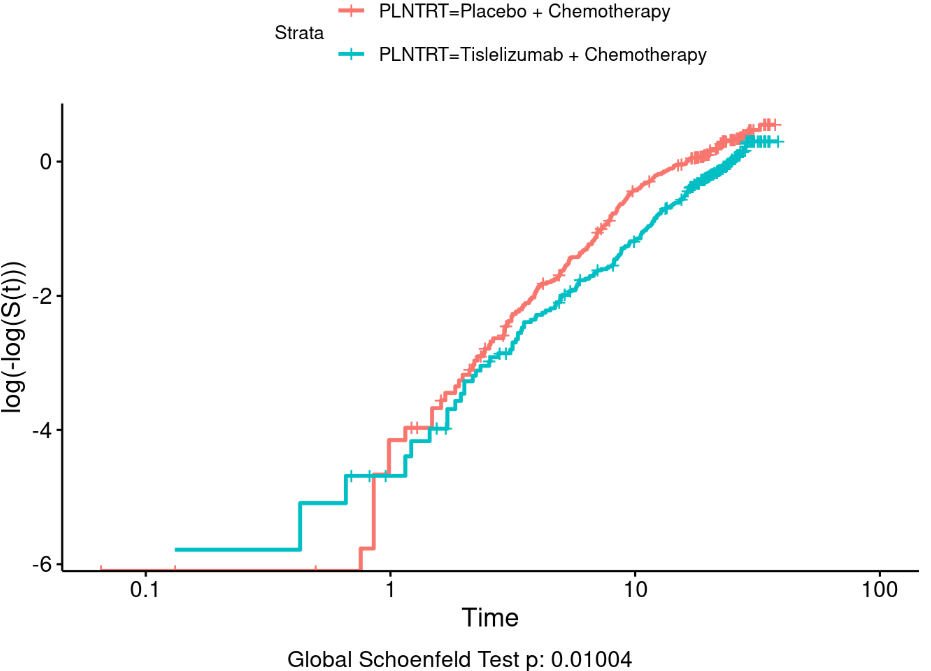


Appendix Figure 15: Schoenfeld residual plots with Grambsch–Therneau p-values for OS, ITT population (RATIONALE-306)


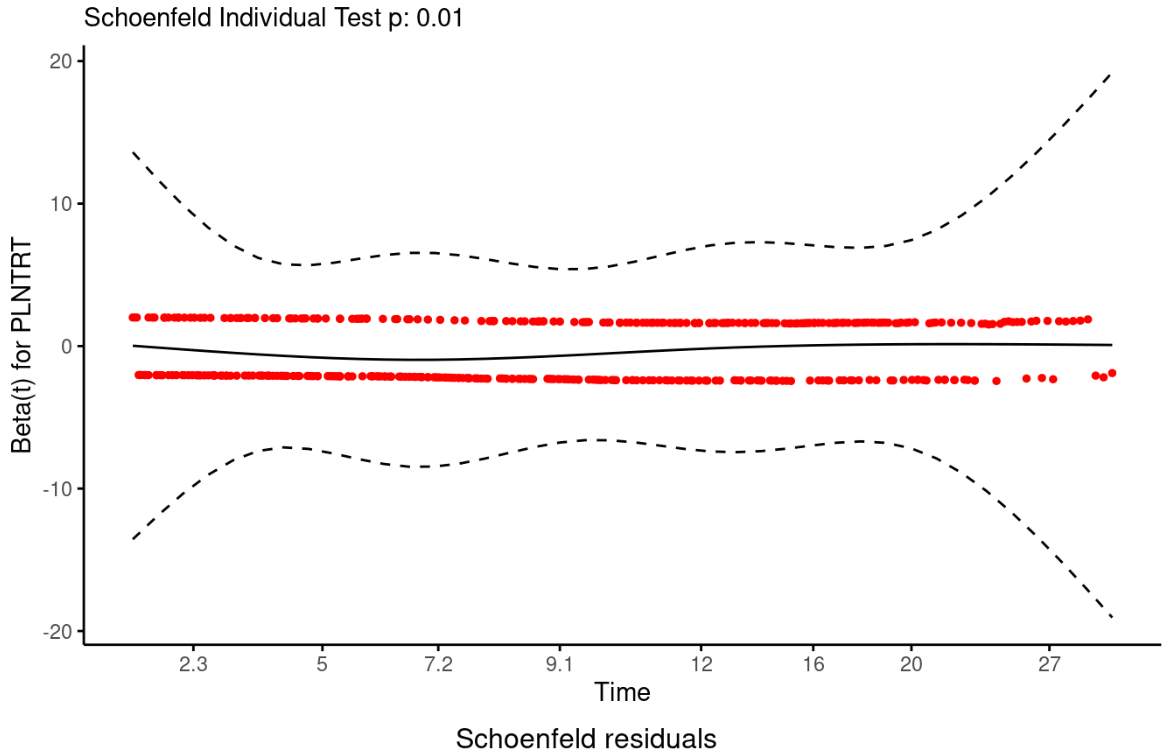


Appendix Figure 16: Cumulative hazard log plots for PFS, ITT population (RATIONALE-306)


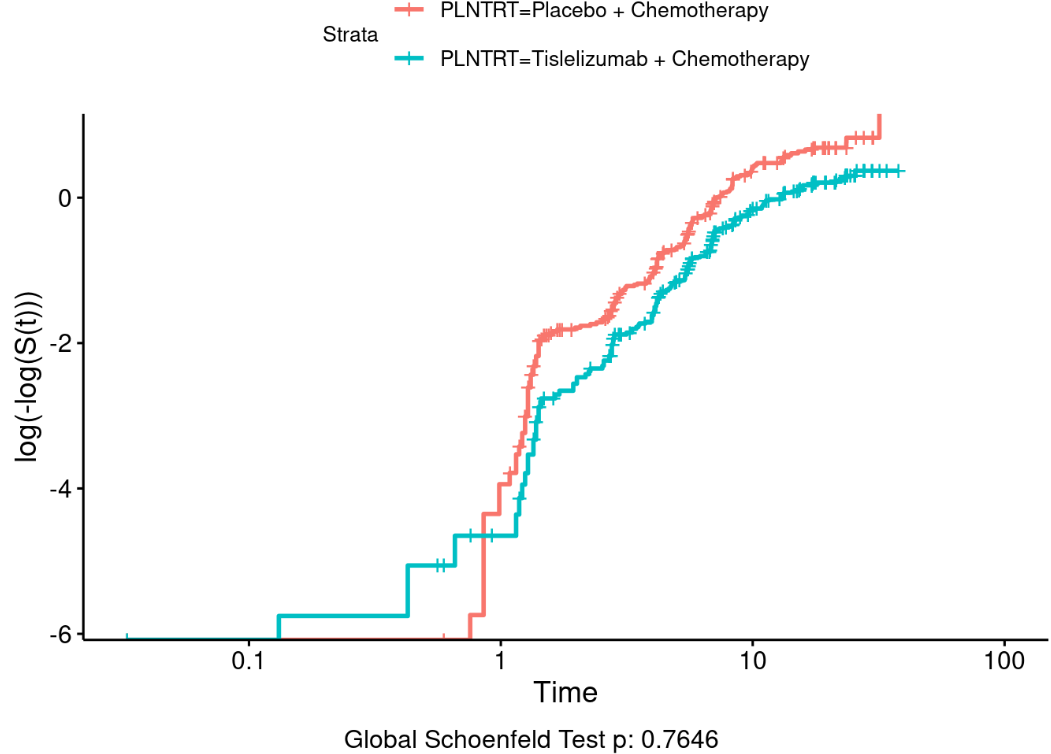


Appendix Figure 17: Schoenfeld residual plots with Grambsch–Therneau p-values for PFS, ITT population (RATIONALE-306)


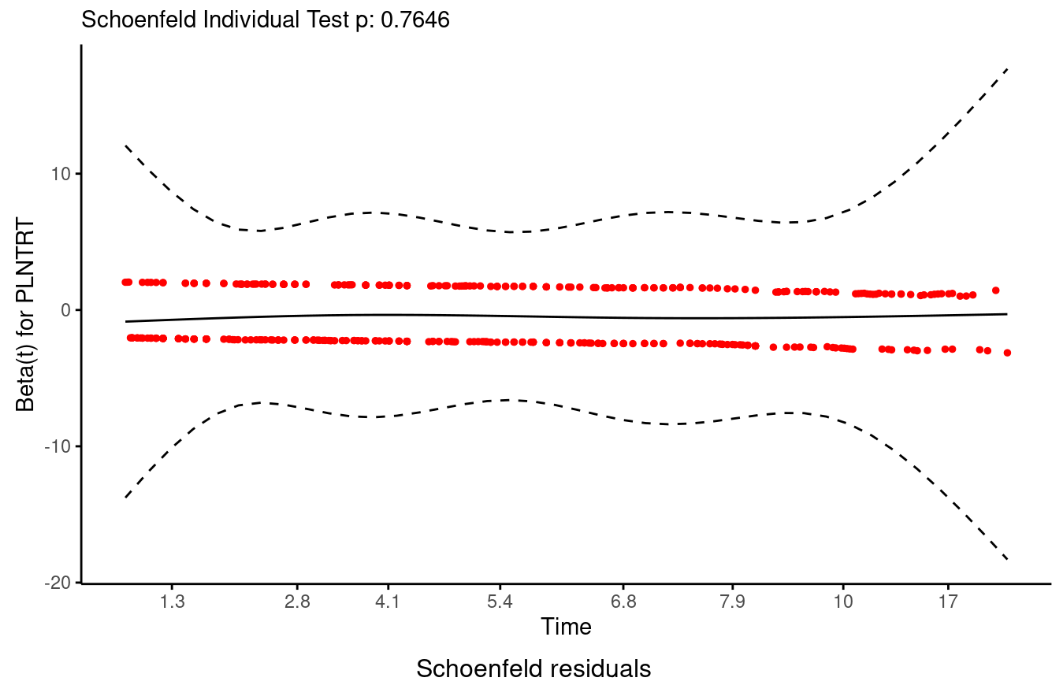

Supplement: Supplementary file 1 [file DataSheet1.docx]
